# Inferring the global structure of chromosomes from structural variations

**DOI:** 10.1186/1471-2164-16-S2-S13

**Published:** 2015-01-21

**Authors:** Tomohiro Yasuda, Satoru Miyano

**Affiliations:** 1 The Human Genome Center, Institute of Medical Science, University of Tokyo, Shiroganedai, Minato-ku, Tokyo, JP; 2 Department of Computer Science, University of Tokyo, Hongo, Bunkyo-ku, Tokyo, JP

## Abstract

**Background:**

Next generation sequencing (NGS) technologies have made it possible to exhaustively detect structural variations (SVs) in genomes. Although various methods for detecting SVs have been developed, the global structure of chromosomes, i.e., how segments in a reference genome are extracted and ordered in an unknown target genome, cannot be inferred by detecting only individual SVs.

**Results:**

Here, we formulate the problem of inferring the global structure of chromosomes from SVs as an optimization problem on a bidirected graph. This problem takes into account the aberrant adjacencies of genomic regions, the copy numbers, and the number and length of chromosomes. Although the problem is NP-complete, we propose its polynomial-time solvable variation by restricting instances of the problem using a biologically meaningful condition, which we call the *weakly connected constraint*. We also explain how to obtain experimental data that satisfies the weakly connected constraint.

**Conclusion:**

Our results establish a theoretical foundation for the development of practical computational tools that could be used to infer the global structure of chromosomes based on SVs. The computational complexity of the inference can be reduced by detecting the segments of the reference genome at the ends of the chromosomes of the target genome and also the segments that are known to exist in the target genome.

## Background

Next-generation sequencing (NGS) technologies have drastically reduced the cost of genome sequencing [1]. As more genomic sequences have become available, it has become clear that genomes contain many *structural variations (SVs)*, which include large insertions, deletions, tandem duplications, and translocations. SVs have already been associated with diverse diseases [2]. For example, the fusion genes BCRABL and EML4-ALK play key roles in the development of cancer, and it is believed that other recurrent rearrangements remain to be discovered [3]. In cancer genomes, many SVs are occasionally concentrated in a small region of the genome [4-6]. It has been suggested that a single catastrophic mutational event, known as *chromothripsis *[6], causes these concentrations. A study of prostate cancer also uncovered a distinct type of complex rearrangement termed *chromoplexy *[7,8], wherein rearrangements are unclustered but involve multiple chromosomes. Complex genomic rearrangements have even been observed in germline mutations, resulting in serious congenital diseases [9]. Because of their importance in functions of the genome, various methods have been developed for finding SVs [10-16]. When genomic rearrangements are complex, enumerating only individual SVs is insufficient for elucidating the *global structure of chromosomes*, i.e., how the segments in a reference genome are extracted and ordered in an unknown target genome. Here, the *reference genome *is known and is a pre-existing sequenced genome of the same organism, such as the GRCh38 build of the human genome [17].

In this study, we address the problem of inferring the global structure of chromosomes based on *SV data*, which refer to aberrant adjacencies of genomic regions and copy number variations (CNVs) in this study. By solving this problem, we can determine the order of the genomic regions in the target genome. This order affects the structure of proteins if the genomic regions contain coding regions, and regulation of genes if the genomic regions include promoters or enhancers. In addition, raw SV data could be corrected by inferring the global structure of chromosomes because an optimal global structure would ignore false positive detection of aberrant adjacencies or correct wrongly estimated copy numbers. The task of inferring chromosomes is formulated as an optimization problem on a graph, which we term as a *chromosome graph*. Our contributions are summarized as follows:

• To infer the global structure of chromosomes, we formulate a computational problem that takes into account the number and length of chromosomes, as well as aberrant adjacencies and CNVs caused by genomic rearrangements. By taking SV data as the input, relatively low-depth NGS sequencing can be used.

• We prove that the problem is NP-complete.

• We propose a biologically meaningful restriction that makes the problem solvable in polynomial time. We also show an algorithm that solves the restricted problem.

Oesper et al. [18] presented a pioneering work that aimed to infer the global structure of chromosomes from SV data. They formulated the *copy number and adjacency genome reconstruction problem*. Their formulation is based on graphs that they termed *interval-adjacency graphs*. These graphs are essentially the same as our chromosome graphs, except that we used bidirected graphs [19,20] while they used alternating paths to exclude paths on the graph that do not correspond to chromosomes. They also implemented an efficient algorithm called *paired-end reconstruction of genome organization (PREGO) *that solved their problem and obtained promising results. Our work includes the following results that were not addressed by Oesper et al. First, we present a formulation that takes into account the number and length of chromosomes determined experimentally. Second, we prove that the problem is NP-complete. Finally, we propose a variation of the problem that can be solved in polynomial time.

Some methods can also be applied to analyze the global structure of genomes by using non-SV data. First, *de novo *sequence assembly aims at reconstructing target genomes from raw NGS sequences [19,21-25]. It includes a step to order fragments of genomes obtained by assembling NGS sequences. The step is usually implemented as an optimization problem, involving searching for paths that cover all vertices or all edges corresponding to substrings of genome sequences [19,21]. By contrast, we allow some vertices and edges to be ignored because some portions of the reference genome might not appear in the target genome. Second, reference-assisted assembly [26], also known as comparative assembly [27], aims at ordering segments of an unknown target genome by using known genomes of other organisms. By contrast, we order segments so that the chromosomes in the solution are most consistent with the SV data and the experimentally determined number and length of chromosomes. Finally, methods based on permutations of integers [28] compare two genomes represented by two sequences of integers corresponding to genes or markers in the genome. Instead of using such sequences, we exploit SV data.

The rest of this paper is organized as follows. First, we present types of experimental data from which we infer the global structure of chromosomes. Next, we give our formulation of the problem of inferring the global structure of chromosomes, and show that the problem is NP-complete. Then, we show a variation of the problem that is solvable in polynomial time. Finally, we discuss our results and state our conclusions.

## Results

### Experimental data

We assume the following experimental data as input.

#### Aberrant adjacencies

**Figure 1:**
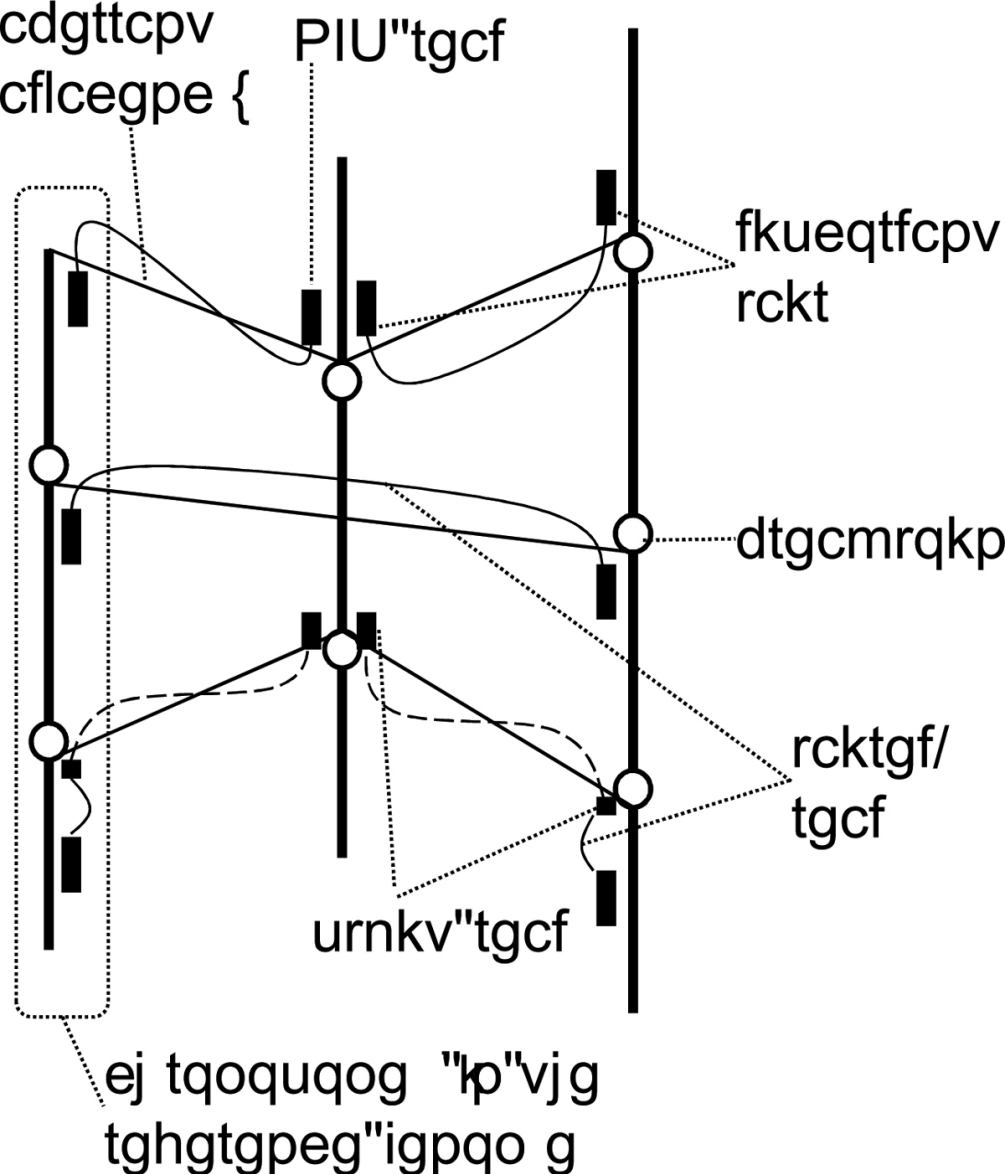
**Aberrant adjacencies of genomic regions**. Thick vertical lines represent chromosomes in the reference genome, circles represent breakpoints, small black boxes represent NGS reads, solid curved lines represent paired-reads, dashed curved lines represent split reads, and thin solid oblique lines represent aberrant adjacencies. Aberrant adjacencies are detected by using two types of NGS reads abnormally mapped to the reference genome: discordant pairs (three pairs from above), and split reads (two pairs from below). Yasuda and Miyano Page 11 of 11

In the target genome, distant segments in the reference genome may be adjacent because of rearrangements (Figure 1). Such aberrant adjacencies are detected by using NGS technologies as follows. First, NGS technologies can generate read pairs that are a few hundred bases apart from each other in the target genome. If two reads of a pair are not mapped to the reference genome with the expected orientations and mapped distance, the pair is called a *discordant pair *and is likely to be caused by SVs [12-14]. Second, if the alignment of a read and reference genome is split into more than one portion, such a split read also indicates a rearrangement [16]. A *breakpoint *is a position at a boundary of a rearrangement. Here, we ignore small differences between the real breakpoints and their estimations.

#### Copy numbers

The number of occurrences of a subsequence in the reference genome may change because of rearrangements. This phenomenon results in *copy number variations (CNVs)*. Traditionally, CNVs have been analyzed by using DNA microarrays [11]. Several recent methods detect CNVs by finding changes in the depth of coverage of NGS sequences [4,15]. Although tumor samples are usually a mixture of normal cells and various tumor cells, the copy numbers of a cancer cell can still be estimated by single-cell analysis [29]. In this paper, for the sake of conciseness, the boundaries of CNVs are also called *breakpoints*.

#### Number of chromosomes and truncations

Identifying chromosomes and finding aberrant chromosomes by microscopy is an important part of clinical diagnostics [30]. The number of chromosomes, denoted by *n_N _*in this paper, is available after inspection. Throughout this paper, we assume that *n_N _*≥ 1. In addition, we also take into account the number of chromosomal truncations, which we denote as *n_T_*. Chromosomal truncations are detected as a decrease in copy numbers without aberrant adjacencies. We consider *n_N _*and *n_T _*to improve the inference of the global structure of chromosomes from SV data.

#### Chromosome length

The length of chromosomes can be estimated experimentally from flow karyotyping, and, approximately, from microscopic images [31]. Here, the estimated length is denoted by *λ_i _*for 1 ≤ *i *≤ *N_L_*, where *N_L_*(≥ *n_N_*) is the maximum possible number of chromosomes.

### Problem definition

Any instance of our problem is modeled as a graph that we term a *chromosome graph*. The graph contains elements derived from the reference genome and experimental data. Each vertex corresponds to a location in the reference genome. In addition, each edge corresponds to either a segment in the reference genome, an adjacency of flanking segments in the reference genome, or an aberrant adjacency in the target genome caused by rearrangements.

We assume that the target genome is a set of chromosomes, each of which is a concatenation of segments in the reference genome. Each chromosome in the target genome is represented as a path on the graph, and these paths explain how segments in the reference genome are incorporated into the target genome. The goodness of the estimated target genome is measured by a cost function, and we search for an optimal set of chromosomes that minimizes this cost function.

We first define a graph that contains some of elements described above. Then, we extend the graph to a chromosome graph. Finally, we present the formal definition of the problem.

#### Prototype chromosome graph

**Figure 2:**
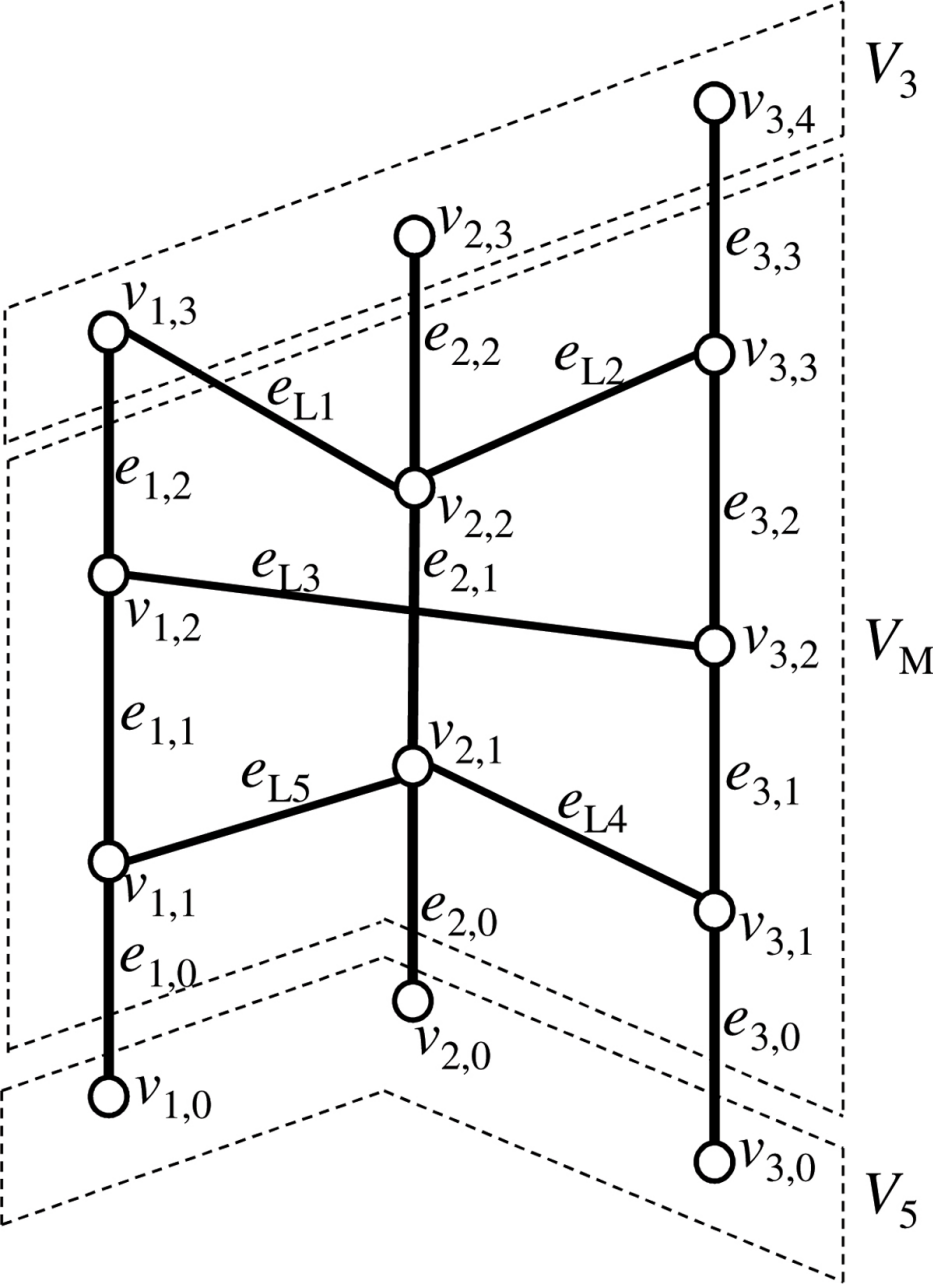
**An example of a prototype chromosome graph**. Thick vertical edges represent edges in *E*_*S *_that correspond to segments in the reference genome, oblique edges represent edges in *E*_*L *_that correspond to aberrant adjacencies. Vertices surrounded by dashed lines belong to *V*_5_, *V*_*M*_, and *V*_3_, read from the bottom of the graph to top.

We first construct an undirected graph called a *prototype chromosome graph*, *G *= (*V,E*) (Figure 2). Let *N_C _*be the number of chromosomes of the reference genome and *n_i _*be the number of breakpoints in the *i*-th chromosome of the reference genome. Then, *V *contains the following vertices.

• Vertices corresponding to breakpoints:

$$V_{M}=\left\{v_{i,j}|1\leq i\leq N_{C},\;1\leq j\leq n_{i}\right\}.$$

• Vertices corresponding to the beginning of chromosomes in the reference genome:

$$V_{5}=\left\{v_{i,0}|1\leq i\leq N_{C}\right\}.$$

• Vertices corresponding to the end of chromosomes in the reference genome:

$$V_{3}=\left\{v_{i,n_{i+1}}|1\leq i\leq N_{C}\right\}.$$

Then, we define *V *= *V*_5 _∪ *V*_3 _∪ *V_M_*.

Next, we define a set of edges, *E*. We make the following two types of edges.

• Edges corresponding to segments between two breakpoints that are next to each other in the reference genome. For each 1 ≤ *i *≤ *N_C _*and 0 ≤ *j *≤ *n_i_*, we make an edge *e_i,j _*= (*v_i,j_*, *v_i,j_*_+1_).

• Edges corresponding to aberrant adjacency of two segments in the reference genome. Let *N_A _*be the number of detected aberrant adjacencies. For the *k*-th aberrant adjacency (1 ≤ *k *≤ *N_A_*) that links positions corresponding to $v_{i_{1},j_{1}}$ and $v_{i_{2},j_{2}}$, we make an edge $e_{Lk}=\left(v_{i_{1},j_{1}},v_{i_{2},j_{2}}\right)$.

Then, we define

$$\begin{array}{c} E_{S}=\left\{e_{i,j}|1\leq i\leq N_{C},\;0\leq j\leq n_{i}\right\},\\ E_{L}=\left\{e_{Lk}|1\leq k\leq N_{A}\right\},\\ \;E=E_{S}\cup E_{L}. \end{array}$$

#### Chromosome graph

**Figure 3:**
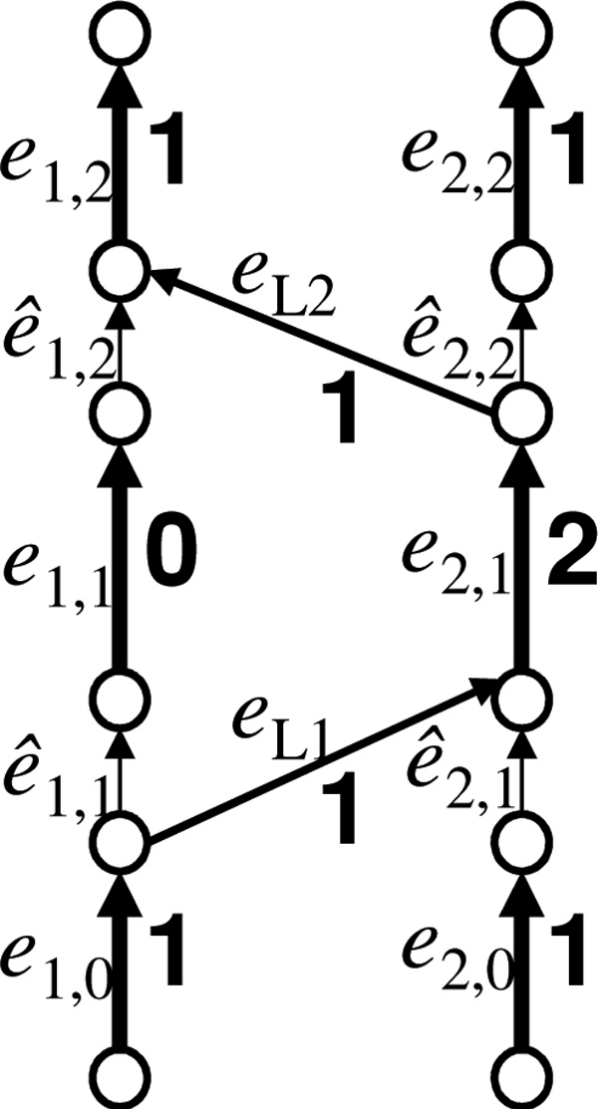
**An example of a chromosome graph**. Thin vertical edges represent edges in *E*_*R*_. Arrowheads represent the '+'-direction, whereas ends of edges without arrowheads represent '−'-direction.

In a prototype chromosome graph, a path might visit two edges in *E_L _*contiguously. Such a path does not correspond to a real chromosome. To exclude such a path we use a technique similar to that of Oesper et al. [18]. Although Oesper et al. [18] used alternating paths, their formulation can be represented by using a bidirected graph whose edges have directions at both ends [19,32]. We directly define our graph by using a bidirected graph (Figure 3). Let *d*(*e*, *v*) ∈ {+,−} be the direction of an edge *e *at a vertex *v*, and −*d*(*e*, *v*) be the opposite direction of *d*(*e*, *v*).

• Each vertex *v_i,j _*∈ *V_M _*is split into two vertices $v_{i,j}^{+}$ and $v_{i,j}^{-}$. The set *V_M _*is redefined as

$$V_{M}=\left\{v_{i,j}^{-},\;v_{i,j}^{+}|1\leq i\leq N_{C},\;1\leq j\leq n_{i}\right\}.$$

Vertices in *V*_5 _and *V*_3 _are renamed so that

$$\begin{array}{c} V_{5}\;=\;\left\{v_{i,0}^{-}|1\leq i\leq N_{C}\right\},\\ V_{3}\;=\;\left\{v_{i,n_{i+1}}^{+}|1\leq i\leq N_{C}\right\}. \end{array}$$

• An edge *e_i,j _*= (*v_i,j_*, *v*_*i,j*+1_) ∈ *E_S _*is reconnected to $v_{i,j}^{-}$ and $v_{i,j+1}^{+}$. In addition, $d\left(e_{i,j},v_{i,j}^{-}\right)=-$ and $d\left(e_{i,j},v_{i,j+1}^{+}\right)=+$.

• Let *e *∈ *E_L _*be an edge connected to *v_i,j _*in the prototype chromosome graph. If *e *corresponds to an aberrant adjacency involving the segment that stretches toward *v*_*i,j*+1_, *e *is reconnected to $v_{i,j}^{-}$ and $d\left(e,v_{i,j}^{-}\right)$ is set to '+'. Otherwise, *e *is reconnected to $v_{i,j}^{+}$ and $d\left(e,v_{i,j}^{+}\right)$ is set to '−'.

• We add the following set of new edges:

$$E_{R}=\left\{{\hat{e}}_{i,j}=\left(v_{i,j}^{+},\;v_{i,j}^{-}\right)|1\leq i\leq N_{C},\;1\leq i\leq n_{i}\right\}.$$

Directions are set so that $d\left({\hat{e}}_{i,j},v_{i,j}^{+}\right)=-$ and $d\left({\hat{e}}_{i,j},v_{i,j}^{-}\right)=+$.

The modified graph represents a *chromosome graph*.

#### Paths and chromosomes

A *path c *= *v*_1_*e*_1_*v*_2_*e*_2_*v*_3 _... *e*_*l*_*v*_*l*+1 _on a chromosome graph *G *is an alternating sequence of vertices and edges, which has the following properties:

• The first and the last of *c *are vertices.

• Any subsequence of the form *e*_*k*_*v*_*k*_*e*_*k*+1 _(1 ≤ *k *≤ *l*) means that *d*(*e_k_*, *v_k_*) = −*d*(*e*_*k*+1_, *v_k_*).

A path *c *is said to *visit *an edge *e *if *c *contains *e*. Similarly, *c *is said to *visit *a vertex *v *if *c *contains *v*. When a path is written as a sequence of vertices and edges, for simplicity, we omit the notation of the vertices if they are clear. Let *C *= {*c*_1_, *c*_2_,..., *c*_|*C*|_} be a multiset of paths on *G*. We define *C *as a multi-set so that more than one identical path can exist. In addition, let *m*(*c*, *e*) be the number of times *c *visits an edge *e*, and $m\left(C,e\right)=\sum_{c_{i}\in C}m\left(c_{i},e\right)$. A *cycle *is a path whose first and last vertices are identical and the directions of the first and the last edges at the vertex are opposite. A *chromosome *on *G *is a path whose first and last edges are both in *E_S_*.

#### Copy numbers and lengths

Two integers are assigned to each *e *∈ *E*. First, *n*(*e*) for *e *∈ *E_S _*represents an experimentally estimated copy number of the corresponding segment in the reference genome. Second, |*e*| for *e *∈ *E_S _*represents the length of the corresponding segment in the reference genome. For *e *∈ *E_L _*∪ *E_R_*, we set *n*(*e*) and |*e*| to 0. The length of a path *c *is defined as $|c|\;=\sum_{e\in E}|e|m\left(c,e\right)$. To simply describe all properties of *e *together, we use the following notation:

$$e=\left\langle d\left(e,\;v_{1}\right)v_{1},\;d\left(e,\;v_{2}\right)v_{2},\;n\left(e\right),\;|e|\right\rangle .$$

#### Upper bound on parameters

Campbell et al. [4] presented examples of amplified regions in cancer cells. The copy numbers were less than 100 in these regions. Therefore, we assume that the copy numbers are in at most hundreds. We also assume that short repeat elements are masked in advance in order to exclude segments that appear spuriously. Based on the details given above, we assume that *n_N_*, *n_T_*, and *n*(*e*) for *e *∈ *E_S _*are all less than a fixed constant *U*. The value of *U *does not have to be determined because *U *is only used in the analysis of computational complexity.

#### Formulation of the problem

To find an optimal set of chromosomes, we define an optimization problem over a chromosome graph. We define a cost function to be used as a target function of the optimization problem. This function imposes costs on the number of chromosomes, the number of chromosomal truncations, and the number of visits to edges, penalizing for deviations from those that are experimentally expected.

Let *C *= {*c*_1_, *c*_2_,..., *c*_|*C*|_} be a multi-set of chromosomes on *G*, and *w_N_*(*C*) be the cost of the difference between *n_N _*and |*C*|. Also let Tr(*C*) be the number of ends of chromosomes in *V_M_*, and *w_T _*(*C*) be the cost of the difference between *n_T _*and Tr(*C*). In addition, *w*(*e*, *x*) for *e *∈ *E_S _*is defined as the cost when *e *is visited *x*-times. For *e *∈ *E_L _*∪ *E_R_*, *w*(*e*, *x*) is set to 0.

We assume that *w_N_*(*C*), *w_T_*(*C*), and *w*(*e*, *x*) for *e *∈ *E_S _*monotonically increase as ||*C*| − *n_N_*|, |Tr(*C*) − *n_N_*|, and |*x *− *n*(*e*)| increase, respectively. Then, we define the cost function *W*(*C*) as follows:

(1) $$W\left(C\right)=w_{N}\left(C\right)+w_{T}\left(C\right)+\underset{e\in E}{\sum }w\left(e,\;m\left(C,\;e\right)\right).$$

We assume that each term is 0 if and only if

(2) $$\left(\begin{array}{c} |C|\;=\;n_{N},\\ Tr\left(C\right)=n_{T},\\ m\left(C,\;e\right)=n\left(e\right)\text{ for }e\in E_{S}. \end{array}\right\}$$

With these notations, we formulate the problem of inferring the global structure of chromosomes as follows:

**Definition 1 **(Chromosome problem (ChrP)) *Suppose that we are given a chromosome graph G *= (*V,E*), *a cost function W*(*C*), *and parameters λ_i _(*1 ≤ *i *≤ *N_L_)*, *where N_L _is the maximum possible number of chromosomes. Then*, *find a multi-set of chromosomes C on G that minimizes W*(*C*) *under the constraint that *|*c_i_*| ≤ *λ_i _for c_i _*∈ *C*.

Although a similar problem was proposed previously [18], its computational complexity was not analyzed.

**Theorem 1 ***ChrP is NP-complete*.

In the Methods section, we prove Theorem 1.

### Polynomial-time solvable variation

We propose a variation of ChrP that is solvable in polynomial time. For *e *∈ *E_L _*∪ *E_R_*, it is highly likely that *m*(*C*, *e*) ≥ 1 if *e *is supported by a large number of paired-reads. Therefore, it is worth considering a variation in which some edges in *E_L_*∪ *E_R _*must appear in the target genome. We refer to the edges as *required edges*. In addition, because chromosomal truncations can be detected, it is also worth considering a variation in which we know where the ends of the chromosomes of the target genome exist in the reference genome. Because the definition of *W*(*C*) is abstract, we focus on a cost function such that

(3) $$\left(\begin{array}{c} w_{N}\left(C\right)=Q_{N}||C|-n_{N}|,\\ w_{T}\left(C\right)=Q_{T}|Tr\left(C\right)-n_{T}|,\\ w\left(e,\;x\right)=|e||x-n\left(e\right)|, \end{array}\right\}$$

where *Q_N _*and *Q_T _*are constants given as parameters. The values of *Q_N _*and *Q_T _*are tuned in advance so that known global structures of genomes are well reconstructed.

#### Weakly connected constraint

Let *G *= (*V*, *E*) be a general bidirected graph. A subgraph *g *of *G *is a *weakly connected component *if *g *is a connected component when all directions are removed [33]. In addition, *g *is *maximal *if *g *is not a subgraph of a larger weakly connected component. For a subset *E' *of *E*, we define CC(*G,E'*) as a set of maximal weakly connected components of a graph induced from *G *by removing the edges not in *E'*.

**Definition 2 **(Weakly connected constraint (WCC)) *Let G *= (*V*, *E*) *be a chromosome graph. Also let V_W _and E_W _be subsets of V and E*, *respectively. Each g *∈ *CC*(*G*, *E_W_*) *is *good *if g contains at least one vertex in V_W_. Then*, *G satisfies the *weakly connected constraint (WCC) *if all g *∈ *CC*(*G*, *E_W_*) *are good*.

**Figure 4:**
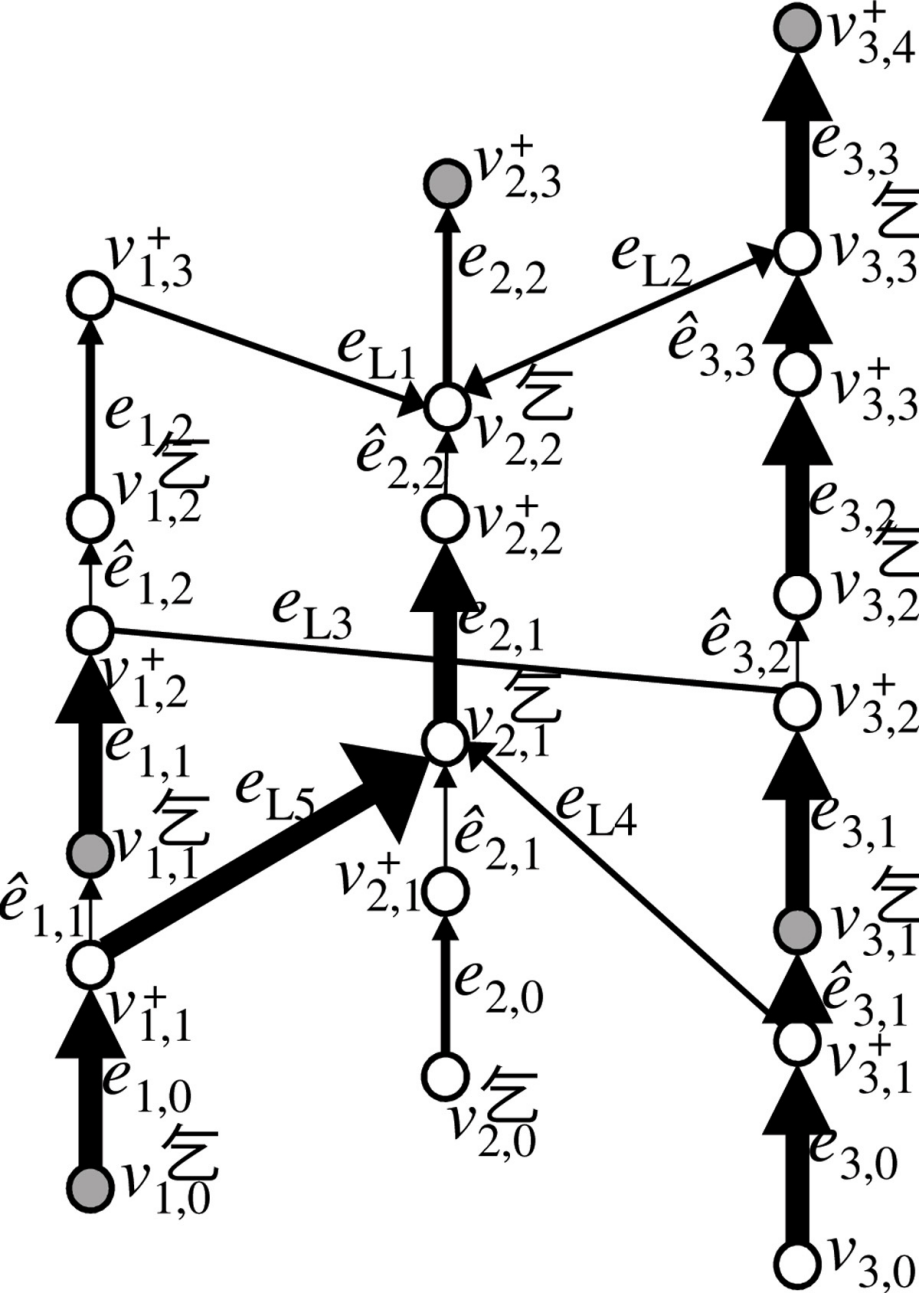
**An example of a chromosome graph that satisfies WCC**. Gray circles are vertices in *V*_W _and thick arrows are edges in *E*_*W*_.

We use WCC by setting *V_W _*to a set of vertices that correspond to ends of chromosomes in the target genome, and *E_W _*= {*e *∈ *E_S_*|*n*(*e*) ≥ 1} ∪ {*e *∈ *E_L _*∪ *E_R_*|*e *is required}. See Figure 4 for an example. An instance that satisfies WCC can be obtained as follows. First, *V_W _*is obtained by finding the positions of chromosomal truncations, as well as the ends of the chromosomes of the reference genome that remain in the target genome. Because a chromosome that does not include detected ends can be in a solution, *V_W _*does not need to contain all ends of chromosomes in the target genome. We assume that *n_T _*≥ |*V_W_*|. Next, if *g *∈ CC(*G*, *E_W_*) is not good, edges *e *∈ *E *on some path connecting *g *and good *g' *∈ CC(*G*, *E_W_*) are added to *E_W_*. To do this, if possible, we experimentally confirm that *n*(*e*) ≥ 1 if *e *∈ *E_S _*or that *e *is required if *e *∈ *E_L _*∪ *E_R_*. Finally, if some *g *∈ CC(*G,E_W_*) that are not good still remain, edges in *g *are forcibly removed from *E_W _*by setting *n*(*e*) to 0 if *e *∈ *E_S _*or by changing *e *not required if *e *∈ *E_L _*∪ *E_R_*.

**Definition 3 **(Chromosome problem with WCC (ChrW)) *Let G *= (*V,E*) *be a chromosome graph that satisfies WCC with respect to some V_W _*⊂ *V and E_W _*⊂ *E. Then*, *find a set C of chromosomes on G that minimizes W*(*C*) *when (3) is satisfied*.

**Theorem 2 ***The problem ChrW can be solved in O*(|*E*|_2 _log |*V *| log |*E*|) *time*.

See the Methods section for the algorithm that solves ChrW.

### Restriction on the length of chromosomes

In ChrW, we removed restrictions on the length of chromosomes. This relaxation is necessary to make the problem solvable in polynomial time.

**Definition 4 **(ChrW with restriction on length (ChrL)) *ChrW with restriction on length (ChrL) is the same problem as ChrW*, *except that the length of each chromosome c_i _is bounded by a parameter λ_i _(*1 ≤ *i *≤ *N_L_)*, *where N_L _is the maximum possible number of chromosomes*.

**Theorem 3 ***The problem ChrL is NP-complete*.

See the Methods section for proof that problem ChrL is NP-complete.

## Discussion

### Handling practical situations

Solutions to the chromosome problems are affected by errors in given SV data. However, some errors can be mitigated as follows. First, a false positive aberrant adjacency may be correctly ignored in the optimal solution because a set of chromosomes that uses such an adjacency is expected to have a larger cost than those ignoring the adjacency. Second, the effects of a missing aberrant adjacency may be limited to segments including its ends because a chromosome that contains the missing adjacency may be recognized as two split chromosomes. Finally, there is a chance that incorrect copy numbers will be corrected if they are inconsistent with other SVs.

In addition to segments in the reference genome, our method can handle newly inserted fragments not in the reference genome. Such a fragment is incorporated Yasuda and Miyano Page 6 of 11 into a chromosome graph as a new chromosome. In particular, an edge *e*, where |*e*| is equal to the length of the fragment, is added to *E_S_*, and edges that connect vertices in a chromosome graph to *e *are added to *E_L_*. If any breakpoints are contained within the new fragment, vertices and edges are added to *V_M _*and *E_R_*, respectively. If a breakpoint corresponds to any aberrant adjacency, edges are also added to *E_L_*.

If a gene duplication has occurred in the target genome, it causes an increased copy number and aberrant adjacencies flanking the gene. If it is a tandem duplication, an aberrant adjacency connecting the upstream and downstream regions of the gene should exist. If these SVs exist in given SV data, any solution to our problem has to take into account gene duplication.

### Limitations

A mixture of many cells cannot be handled because it is difficult to correctly estimate copy numbers. However, our method may generate meaningful results for data obtained from multiple cells if the sum of copy numbers is correctly estimated. In this case, the solution is a mixture of chromosomes of all cells in the sample, although some of the chromosomes might be fused.

**Figure 5:**
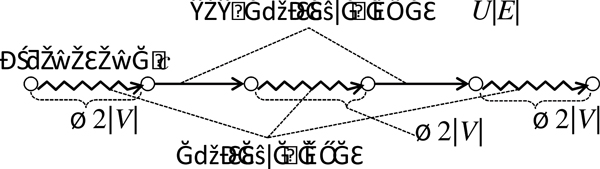
**An example of a chromosome graph that has more than one optimal solution**. Bold digits represent an optimal circulation on this graph. The chromosome graph in this figure has two optimal solutions {*e*_1, 0_*e*_L1_*e*_2, 1_*e*_L2_*e*_1,2_, *e*_2,0_*ê*_2,1_*e*_2,1_*ê*_2, 2_*e*_2,2_} and {*e*_1,0_*e*_L1_*e*_2, 1_*ê*_2,2_*e*_2,2_, *e*_2,0_*ê*_2,1_*e*_2,1_*e*_L2_*e*_1,2_}. Edges in *E*_*N *_∪ *E*_*D *_are omitted, and the flow on each edge in *E*_*D *_has been subtracted from the flow of a corresponding edge in *E*_*S*_.

Note that many optimal solutions may exist depending on how an optimal circulation is converted into chromosomes. (Figure 5). Choosing the right solution requires additional information such as the mate-pairs of long genomic fragments, or the result of experiments involving such techniques as fluorescence *in situ *hybridization (FISH) that indicate whether or not distant genomic regions are in the same chromosome.

### Toward implementation

For implementation, we require an algorithm that can calculate an optimal circulation on the bidirected graph. It would be difficult to implement Gabow's algorithm because no efficient implementation is currently known. Another option would be to use Medvedev's algorithm [19]. Any solver for general integer programming could also be used, as demonstrated by Oesper et al. [18], although the computational time bound is not guaranteed.

## Conclusions

Continuing technological innovations in DNA sequencing will, in future, allow the prediction of an enormous number of SVs. However, detecting only individual SVs cannot reveal the global structure of chromosomes. Here, we formulated the problem of inferring chromosomes from the aberrant adjacencies of genomic regions, copy number variations (CNVs), and the number and length of chromosomes. The problem, which we term as the *chromosome problem (ChrP)*, was proved to be NP-complete. However, if an instance of ChrP satisfies a constraint, which we call a *weakly connected constraint (WCC)*, and if the length of chromosomes is ignored, the problem can be solved in *O*(|*E*|^2 ^log |*V *| log |*E*|) time.

This work provides a theoretical basis for the development of practical computational tools that are emerging for use in analysis of the global structure of chromosomes based on SVs.

## Methods

In this section, we show how we proved the theorems stated in the Results section.

### Proof of Theorem 1

We first present an upper bound on the size of an optimal solution of ChrP to show that ChrP is in NP. Then, we prove that ChrP is NP-hard.

**Lemma 1 ***Let G *= (*V*, *E*) *be a chromosome graph. Also*, *let C be a multi-set of chromosomes on G that minimizes W*(*C*) *such that *|*c_i_*| ≤ *λ_i _for c_i _*∈ *C. Then*, *C has at most U*(4|*V*| + 1)(|*E*| + 1) *edges*.

**Figure 6:**
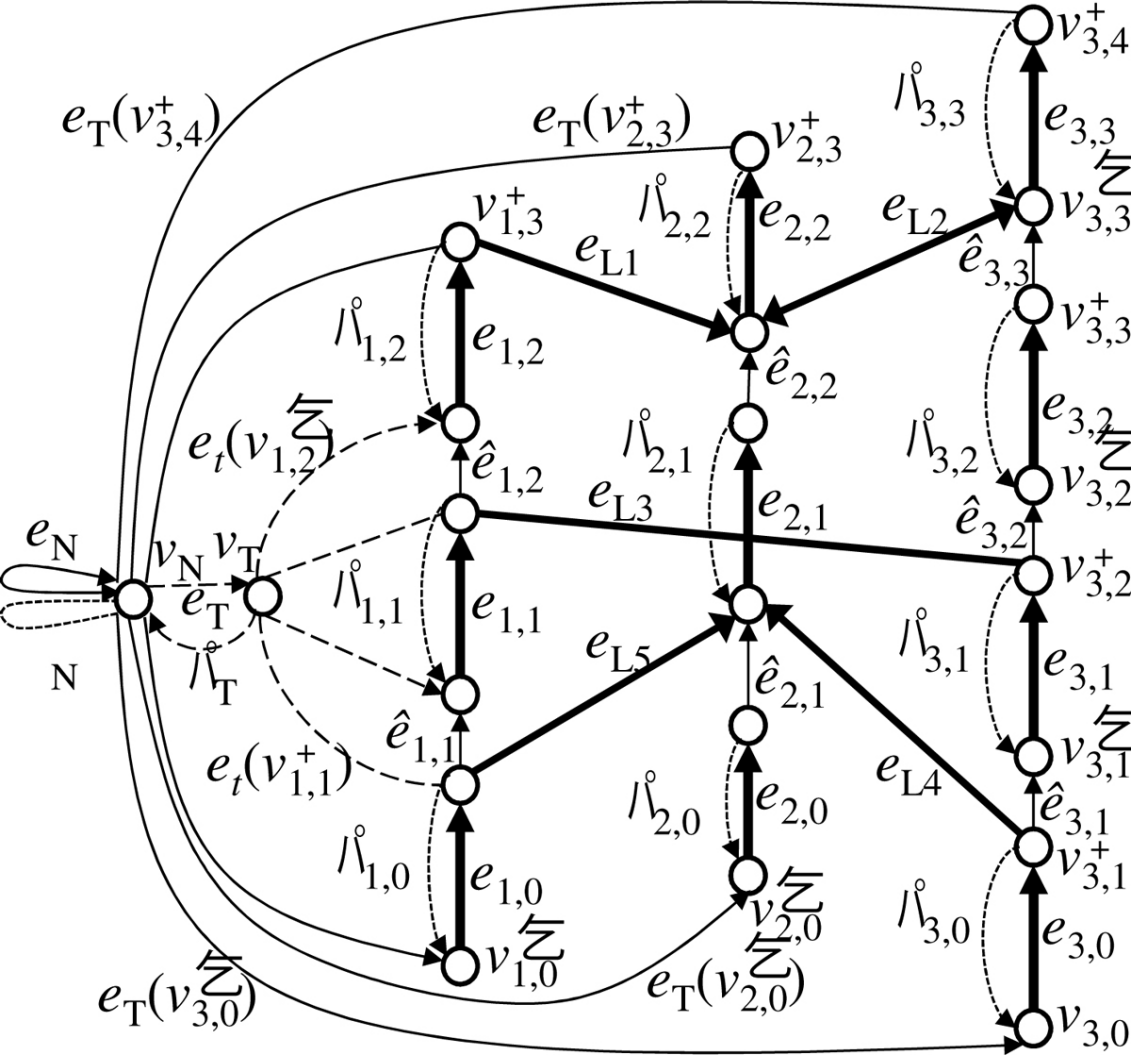
**An example of a chromosome that consists of non-excessive and excessive edges**. Straight arrows represent non-excessive edges, while jagged lines represent sequences of excessive edges.

*Proof *Let *c *∈ *C *be a chromosome in *C*. We define an edge *e *in *c *as *non-excessive *if *e *∈ *E_S _*and *m*(*C*, *e*) ≤ *n*(*e*), and *excessive *otherwise. Let *t_c _*be the number of non-excessive edges visited by *c*. If *t_c _>*0, *c *can be written as *c *= *p*_1_*e*_1_*p*_2_*e*_2 _... *e_t_*c*p_t_*c_+1_, where *e_k _*(1 ≤ *k *≤ *t_c_*) is a non-excessive edge and *p_k _*(1 ≤ *k *≤ *t_c_*+1) is a possibly empty path that contains only excessive edges (Figure 6). If *p_k _*contains a cycle as its subpath, the cycle can be removed to decrease *W*(*C*), a contradiction. Accordingly, *p_k _*does not contain a cycle. This implies that *p_k _*visits at most 2|*V*| vertices and, thus, 2|*V*| edges. Therefore, at most, 4|*V *| excessive edges are visited for each non-excessive edge. Note that a non-excessive edge *e *can be visited, at most, *n*(*e*)-times. Therefore, $\sum_{c\in C}t_{c}\leq \sum_{e\in Es}n\left(e\right)$.

Chromosomes such that *t_c _*= 0 can exist only if they contribute to the decrease of the first or the second term of *W*(*C*) defined by (1). Accordingly, the number of such chromosomes is, at most, *n_N _*+*n_T_*. In addition, a chromosome *c*, such that *t_c _*= 0, does not contain any cycles because such a cycle can be removed to decrease *W*(*C*). Therefore, at most, *c *visits 2|*V*| vertices and, thus, 2|*V*| edges.

Consequently, *C *contains, at most, 2|*V*|(*n_N _*+*n_T _*) + (4|*V*| +1) P_e_***_E_S *n*(*e*) ≤ *U*(4|*V *|+1)(|*E*|+1) edges.

**Lemma 2 ***The problem ChrP is in NP*.

*Proof *Once an optimal solution *C *is given, whether or not *W*(*C*) is greater than a given constant can be determined in *O*(|*V *||*E*|) time by Lemma 1.   □

**Lemma 3 ***The problem ChrP is NP-hard*.

**Figure 7:**
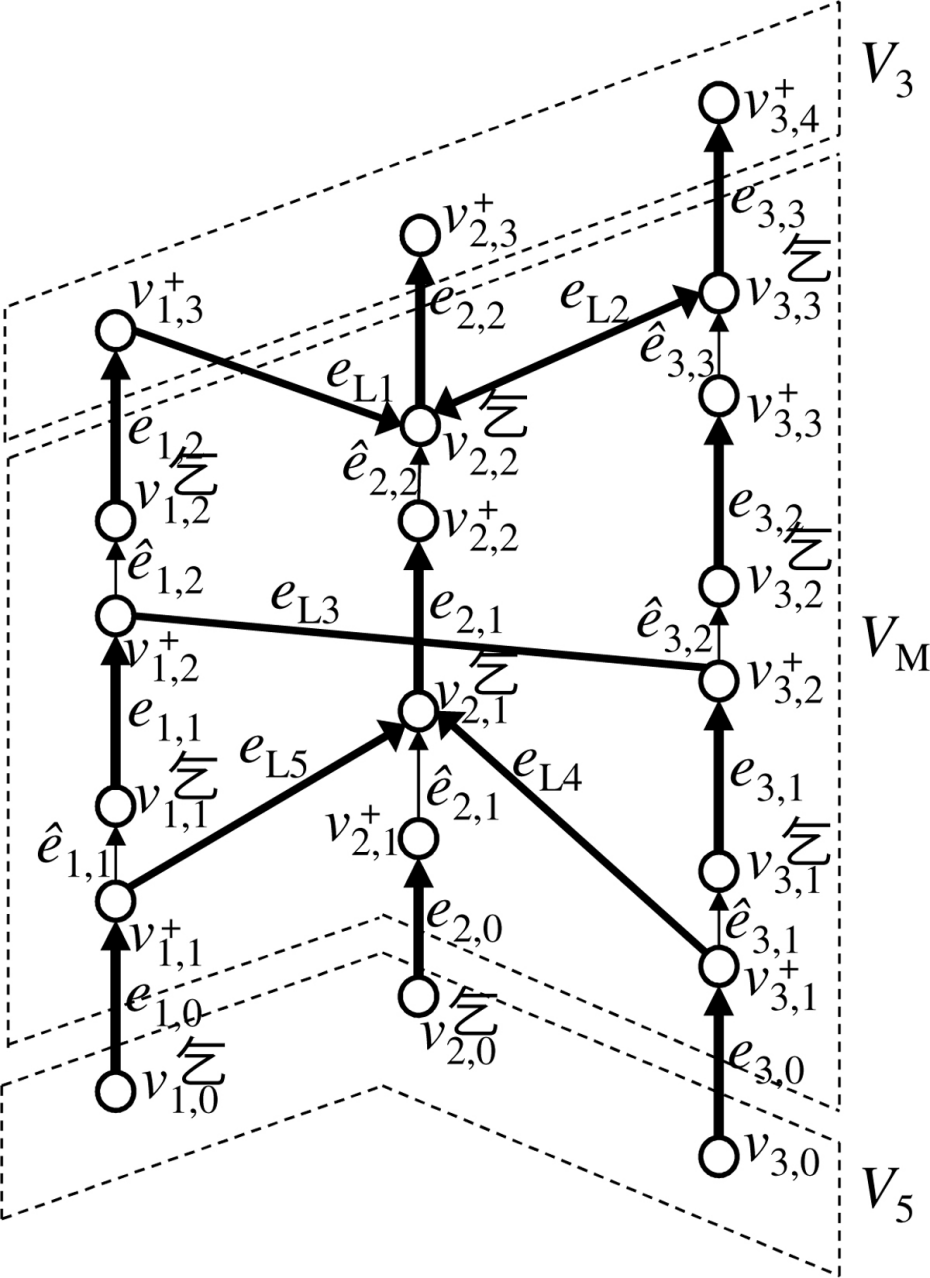
**An instance of ChrP for solving the Hamiltonian Cycle problem (HC)**. In this graph, solid edges are constructed for each vertex in a graph *H *of HC, whereas dashed edges correspond to edges in *H*.

*Proof *The *Hamiltonian Cycle problem (HC) *is a problem of finding a cycle that visits each vertex of a graph exactly once, and is a well-known NP-complete problem [34]. Here, we reduce HC to ChrP. Consider HC on a directed graph *H *= (*V'*, *E'*), where $V^{\prime }=\left\{v_{1}^{\prime },v_{2}^{\prime },\ldots ,v_{\left|V^{\prime }\right|}^{\prime }\right\}$ is a set of vertices and *E' *is a set of edges. We construct a chromosome graph *G *= (*V*, *E*) from *H *(Figure 7), where

$$V=\underset{1\leq i\leq |V^{\prime }|}{⋃}\left\{v_{i,0}^{-},\;v_{i,1}^{+},\;v_{i,1}^{-},\;v_{i,2}^{+},\;v_{i,2}^{-},\;v_{i,3}^{+}\right\}$$

is a set of vertices, and *E *= *E_S _*∪ *E_L _*∪ *E_R _*is a set of edges. Here, *E_S _*consists of

$$\begin{array}{c} e_{1,0}=\left\langle -v_{1,0}^{-},+v_{1,1}^{+},1,1\right\rangle ,\\ e_{1,1}=\left\langle -v_{1,1}^{-},+v_{1,2}^{+},2,1\right\rangle ,\\ e_{1,2}=\left\langle -v_{1,2},+v_{1,3}^{+},1,1\right\rangle ,\\ e_{i,0}=\left\langle -v_{i,0},+v_{i1}^{+},0,1\right\rangle \;\left(2\leq i\leq |V^{\prime }|\right),\\ e_{i,1}=\left\langle -v_{i,1}^{-},+v_{i2}^{‡},1,1\right\rangle \;\left(2\leq i\leq |V^{\prime }|\right),\\ e_{i,2}=\left\langle -v_{i,2}^{-},+v_{i,3}^{‡},0,1\right\rangle \;\left(2\leq i\leq |V^{\prime }|\right). \end{array}$$

*E_R _*consists of

$$\begin{array}{c} {\hat{e}}_{i,1}=\left\langle -v_{i,1}^{+},+v_{i,1}^{-},0,0\right\rangle \left(1\;\leq i\leq |V^{\prime }|\right),\\ {\hat{e}}_{i,2}=\left\langle -v_{i,2}^{+},+v_{i,2}^{-},0,0\right\rangle \left(1\;\leq i\leq |V^{\prime }|\right). \end{array}$$

*E_L _*consists of

$$e_{i^{\prime }:i}=\left\langle -v_{i,2}^{+},+v_{i,1}^{-},0,0\right\rangle \;\left(\left(v_{i}^{\prime },\;v_{i}^{\prime }\right)\in E^{\prime }\right).$$

In addition, we set *n_N _*= 1, *n_T _*= 0, and *λ_i _*= |*V'*| + 3 for any *i*. Then, we prove that *H *has a Hamiltonian cycle if, and only if, ChrP on *G *has a solution *C *such that *W*(*C*) = 0. Suppose that *h *is a Hamiltonian cycle on *H*. Let *c *be a chromosome that begins with $e_{1,0}{\hat{e}}_{1,1}e_{1,1}$ and then visits $e_{i^{\prime }:i}e_{i,1}$ in the order that edges $\left(v_{i^{\prime }},v_{i}\right)$ appear in *h *from *i' *= 1, and finally ends with $e_{1,1}{\hat{e}}_{1,2}e_{1,2}$. Then, a set of a single chromosome *C *= {*c*} satisfies *W*(*C*) = 0 and $|c|=|V^{\prime }|+3\leq \lambda_{1}$.

Conversely, let *C *be a solution of ChrP that satisfies *W*(*C*) = 0. Because (2) holds, |*C*| = 1, Tr(*C*) = 0, and *m*(*C*, *e*) = *n*(*e*). Let *c *be the only chromosome in *C*. Because *n*(*e*_1,1_) = 2 and *n*(*e*_*i*,1_) = 1 for 2 ≤ *i *≤ |*V'*|, a path that visits vertices $v_{i}^{\prime }\in V^{\prime }$ in the order that *e*_*i*,1 _appears in *c *is a Hamiltonian cycle on *H*.   □

Theorem 1 directly follows Lemma 2 and 3.

### Proof of Theorem 2

#### Circulation on a bidirected graph

Let *G *= (*V*, *E*) be a bidirected graph, and *a_v,e _*for *v *∈ *V *and *e *∈ *E *be an integer such that

$$a_{v,e}=\left\{\begin{array}{c} 2\;\text{if }e{\text{ has two}}^{\prime }{+}^{\prime }\text{-ends at }v,\\ 1\;\text{if }e{\text{ has only one}}^{\prime }{+}^{\prime }\text{-end at }v,\\ -1\;\text{if }e{\text{ has only one}}^{\prime }{-}^{\prime }\text{-end at }v,\\ -2\;\text{if }e{\text{ has two}}^{\prime }{-}^{\prime }\text{-ends at }v,\\ 0\;\text{if }e\text{ is not connected to }v. \end{array}\right)$$

Also let *b_v _*be an integer defined for each *v *∈ *V*, *Z *be the set of non-negative integers, and *l*(*e*) and *u*(*e*) be two non-negative integers assigned to each edge *e *∈ *E *called a *lower bound *and an *upper bound*, respectively. Unless otherwise specified, in this study *l*(*e*) = 0 and *u*(*e*) = ∞.

**Definition 5 ***A *bidirected flow (biflow) *[19*, *20] is a mapping f *: *E *→ *Z such that*

(4) l(e)≤f(e)≤u(e)foreache∈E,

(5) $$\underset{e\in E}{\sum }a_{v,e}f\left(e\right)=b_{v}\;for\;each\;v\in V.$$

*The cost of f is defined as W*(*f*) = ∑_*e*∈*E *_*w*(*f*, *e*), *where w*(*f*, *e*) *is a cost of f on e *∈ *E. A *circulation *is a biflow such that b_v _*= 0 *for any v *∈ *V*.

#### Circular chromosome graph

**Definition 6 **(Circular chromosome graph) *Let G *= (*V,E*) *be a chromosome graph*, *and let v_N _and v_T _be new vertices. In addition*, *let E_N _be a set of the following edges: for *1 ≤ *i *≤ *N_C_*,

$$\begin{array}{c} e_{t}\left(v_{i,0}^{-}\right)=\left\langle -v_{N},+v_{i,0}^{-},0,0\right\rangle ,\\ e_{t}\left(v_{i,n_{i}}^{+}\right)=\left\langle -v_{N},-v_{i,n_{i}}^{+},\;0,0\right\rangle ,\\ e_{t}\left(v_{i,j}^{+}\right)=\left\langle -v_{T},-v_{i,j}^{+},\;0,0\right\rangle \;\left(1\;\leq j\leq n_{i}\right),\\ e_{t}\left(v_{i,j}^{-}\right)=\left\langle -v_{T},+v_{i,j}^{-},\;0,0\right\rangle \;\left(1\;\leq j\leq n_{i}\right), \end{array}$$

and

$$\begin{array}{c} e_{T}=\left\langle -v_{N},+v_{T},\;n_{T},\;Q_{T}\right\rangle ,\\ e_{N}=\left\langle +v_{N},+v_{N},\;n_{N},\;Q_{N}\right\rangle . \end{array}$$

*Also*, *let E_D _be a set of the following edges for e *∈ *E_S _*∪ {*e_N_*, *e_T_*}*:*

$$\bar{e}=\left\langle -d\left(e,\;v_{i_{1},j_{1}}\right)v_{i_{1},j_{1}},-d\left(e,\;v_{i_{2},j_{2}}\right)v_{i_{2},j_{2}},0,\;|e|\right\rangle ,$$

*where $v_{i_{1},j_{1}}$ and $v_{i_{2},j_{2}}$ are vertices at the ends of e. The graph *$\tilde{G}=\left(V\cup \left\{v_{N},v_{T}\right\},E\cup E_{N}\cup E_{D}\right)$*is called a *circular chromosome graph.

**Figure 8:**
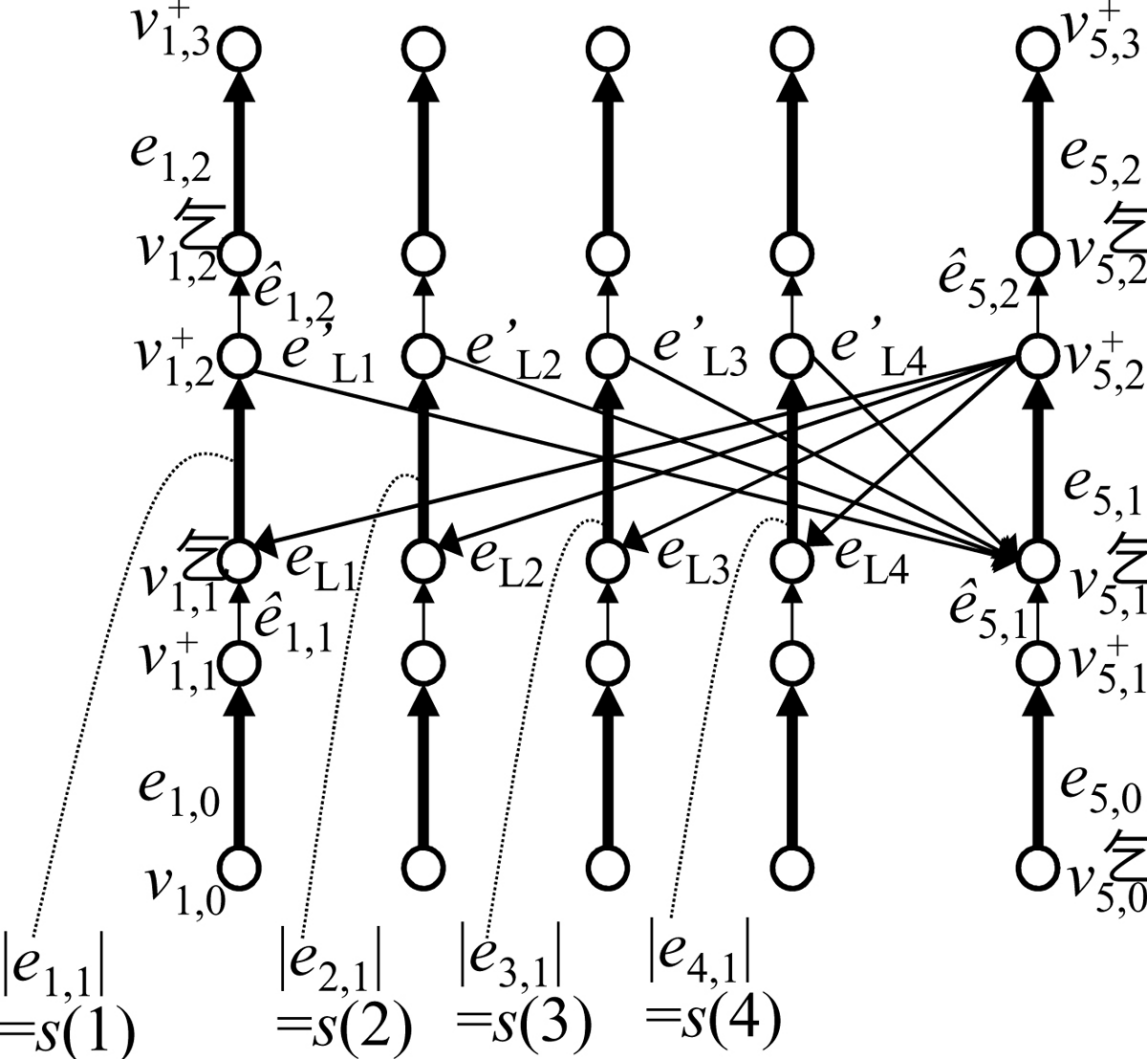
**An example of a circular chromosome graph**. The problem of optimizing multiple chromosomes is converted to the problem of finding a cycle on this graph. For simplicity, we omitted *e*_*t*_(·), except for the leftmost chromosome in the reference genome.

See Figure 8 for an example. Let *n*(*e_N_*) = *n_N _*and *n*(*e_T_*) = *n_T_*. For *e *∈ *E_S _*∪{*e_N_*, *e_T_*}, we set *l*(*e*) = *n*(*e*), l(ē)=0, and u(ē)=n(e). For *e *∈ *E_L _*∪ *E_R_*, we set *l*(*e*) = 1. We also set *l*(*e_t_*(*v*)) to 1 for *v *∈ *V_W _*because these edges have to be visited in the solution.

**Lemma 4 ***Let w*(*f*, *e*) = |*e*|*f*(*e*) *and W*_0 _= *Q_N_n_N _*+ *Q_T _n_T _*+ ∑_*e*∈*E *_|*e*|*n*(*e*)*. For any multi-set C of chromosomes on G*, *there is a circulation f on $\tilde{G}$ such that*

(6) $$W\left(f\right)=W\left(C\right)+W_{0}.$$

*Conversely*, *for any circulation f on $\tilde{G}$that minimizes W(f)*, *there is a multi-set C of chromosomes on G that satisfies (6). In addition*, *C can be calculated in *$O\left(\sum_{e\in E\cup E_{N}\cup E_{D}}f\left(e\right)\right)$*time*.

Let *E*_+ _= {*e *∈ *E *∪ *E_N _*∪ *E_D_*|*l*(*e*) ≥ 1 or *n*(*e*) ≥ 1}. Note that $\text{CC}\left(\tilde{G},E+\right)$ has only one weakly connected component because of WCC.

*Proof *First, we show that for any multi-set *C *of chromosomes on *G*, there exists a circulation *f *on $\tilde{G}$ that satisfies (6). Let End(*v*) be the number of chromosomes that begin or end with *v*. Consider the following *f*:

$$\begin{array}{llll} \;f(e) & = & \max\{n(e),\;m(C,\;e)\} & \;(e\in E_{S}),\\ \;f(\bar{e}) & = & \max\{0,\;n(e)-m(C,\;e)\} & \;(e\in E_{S}),\\ \;f(e) & = & m(C,\;e) & (e\in E_{L}\cup E_{R}),\\ f(e_{t}(v)) & = & \text{End}(v) & (v\in V),\\ \;f(e_{N}) & = & \max\{n_{N},\;|C|\}, & \\ \;f({\bar{e}}_{N}) & = & \max\{0,\;n_{N}-|C|\}, & \\ \;f(e_{T}) & = & \max\{n_{T},\text{Tr}(C)\}, & \\ \;f({\bar{e}}_{T}) & = & \max\{0,\;n_{T}-\text{ Tr}(C)\}. & \end{array}$$

Then, *f *is a circulation on $\tilde{G}$ because *f *satisfies (4) and (5). Thus, we observe that

w(e,m(C,e))=|e|f(e)+|e|f(ē)-|e|n(e),

for *e *∈ *E_S_*, and

$$\begin{array}{c} w_{N}\left(C\right)=|e_{N}|f\left(e_{N}\right)+|e_{N}|f\left({ē}_{N}\right)-Q_{N}n_{N},\\ w_{T}\left(C\right)=|e_{T}|f\left(e_{T}\right)+|e_{T}|f\left({ē}_{T}\right)-Q_{T}n_{T}. \end{array}$$

Therefore, because |*e*| = 0 for *e *∈ *E_L_*∪ *E_R_*∪{*e_t_*(*v*)|*v *∈ *V*} and *w*(*f*, *e*) = |*e*|*f*(*e*), *f *satisfies (6).

Conversely, let *f *be a circulation on $\tilde{G}$ that minimizes *W*(*f*). We show how to construct a multi-set *C *of chromosomes on *G *that satisfies (6).

First, for *e *∈ *E_S _*∪ {*e_N_*, *e_T_*}, we subtract f(ē) from *f*(*e*), and also set f(ē) to 0.

Second, we construct a set *R *of cycles such that *m*(*R*, *e*) = *f*(*e*) for any edge *e *in $\tilde{G}$. For directed graphs, the *flow decomposition theorem *[35] ensures that such *R *can be obtained in $O\left(\sum_{e\in E\cup E_{N}\cup E_{D}}f\left(e\right)\right)$ time. This is also true for bidirected graphs.

Third, we merge cycles in *R*. Whenever a vertex is shared by two cycles in *R*, they are merged into a single cycle. Because of WCC, $\text{CC}\left(\tilde{G},E+\right)$ consists of only one weakly connected component. This implies that all cycles that contain edges in *E*_+ _can be merged into a single cycle. Note that any *r *∈ *R *contains at least one edge in *E*_+_, because otherwise *r *can be removed to decrease *W*(*f*). Therefore, all cycles in *R *can be merged into a single cycle $\tilde{r}$.

Finally, let *C *be a multi-set of paths generated by removal of *v_N_*, *v_T_*, and edges in *E_N _*from $\tilde{r}$. Because *c *∈ *C *is connected to edges in *E_N _*in $\tilde{r}$, the first and last edge of *c *is in *E_S _*due to the directions of these edges. Accordingly, *c *is a chromosome. Therefore, *C *is a multi-set of chromosomes on *G*.

All of these steps can be completed in $O\left(\sum_{e\in E\cup E_{N}\cup E_{D}}f\left(e\right)\right)$ time. In addition, we observe that the following equations hold:

$$\begin{array}{c} |C|\;=\;f\left(e_{N}\right)-f\left({\bar{e}}_{N}\right),\\ \text{Tr}\left(C\right)=f\left(e_{T}\right)-f\left({\bar{e}}_{T}\right),\\ m\left(C,\;e\right)=f\left(e\right)+f\left(\bar{e}\right)\left(e\in E_{S}\right). \end{array}$$

Accordingly, w(e,m(C,e))=w(f,e)+w(f,ē)+|e|n(e) for *e *∈ *E_S_*, and

$$\begin{array}{c} w_{N}\left(C\right)\;=\;w\left(f,\;e_{N}\right)+w\left(f,{\bar{e}}_{N}\right)+Q_{N}n_{N},\\ w_{T}\left(C\right)\;=\;w\left(f,\;e_{T}\right)+w\left(f,{\bar{e}}_{T}\right)+Q_{T}n_{T},\\ w\left(e,\;m\left(C,\;e\right)\right)=0\left(e\in E_{L}\cup E_{R}\right). \end{array}$$

Therefore, *C *satisfies (6).

By Lemma 4, the solution of ChrW can be obtained by calculating a circulation *f *on $\tilde{G}$ that minimizes *W*(*f*). By Lemma 1, setting *u*(*e*) = *U*(4|*V*| + 1)(|*E*| + 1) does not affect the solution. In addition, |*E_N_*| = *O*(|*E*|) and |*E_D_*| = *O*(|*E*|). Accordingly, the circulation *f *can be calculated in *O*(|*E*|_2 _log |*V*| log |*E*|) time by using Gabow's algorithm [20]. Therefore, the optimal solution can be calculated in *O*(|*E*|_2 _log |*V*| log |*E*|) time.

### Proof of Theorem 3

ChrL is in NP because of Lemma 1.

Here, we show that the well-known PARTITION problem [34] can be reduced to ChrL. Let *n *be a positive integer and *S *= {*i *∈ *Z*|1 ≤ *i *≤ *n*}. Also, let *s*(*i*) be an integer function defined for *i *∈ *S *such that Yasuda and Miyano Page 9 of 11 *s*(*i*) > 0, and *S*_Σ _= ∑_*i*∈*S *_*s*(*i*). The problem of finding a subset *S' *⊂ *S *such that

$$\underset{i\in S^{\prime }}{\sum }s\left(i\right)=\underset{i\in S-S^{\prime }}{\sum }s\left(i\right)=S_{\Sigma }/2$$

is called the *partition problem *(hereafter referred to as *PARTITION*) [34]. It is well known that PARTITION is NP-complete. We reduce PARTITION to ChrL by constructing a chromosome graph whose solution for ChrL contains two chromosomes that correspond to two subsets of a solution of PARTITION.

Let *G *= (*V*, *E*) be a chromosome graph, where

$$V=\underset{1\leq i\leq n+1}{⋃}\left\{v_{i,0}^{-},\;v_{i,1}^{+},\;v_{i,1}^{-},\;v_{i,2}^{+},\;v_{i,2}^{-},\;v_{i,3}^{+}\right\}$$

is a set of vertices, and *E *= *E_S _*∪ *E_L _*∪ *E_R _*be a set of edges. Here, *E_S _*consists of

$$\begin{array}{c} e_{i,0}=\left\langle -v_{i,0}^{-},+v_{i,1}^{+},1,9S_{\Sigma }\right\rangle \;\left(1\;\leq i\leq n\right),\\ e_{i,1}=\left\langle -v_{i,1}^{-},+v_{i,2}^{+},2,\;s\left(i\right)\right\rangle \;\left(1\;\leq i\leq n\right)\\ e_{i,2}=\left\langle -v_{i,2}^{-},+v_{i,3}^{+},1,\;S_{\Sigma }-s\left(i\right)\right\rangle \;\left(1\leq i\leq n\right)\\ e_{n+1,0}=\left\langle -v_{n+1,0}^{-},+v_{n+1,1}^{+},2,9S_{\Sigma }/2\right\rangle ,\;\\ e_{n+1,1}=\left\langle -v_{n+1,1}^{-},+v_{n+1,2}^{+},\;n+2,0\right\rangle ,\\ e_{n+1,2}=\left\langle -v_{n+1,2}^{-},+v_{n+1,3}^{+},2,5S_{\Sigma }\right\rangle . \end{array}$$

In addition, *E_R _*consists of

$$\begin{array}{c} {\hat{e}}_{i,1}=\left\langle -v_{i,1}^{+},+v_{i,1}^{-},0,0\right\rangle \;\left(1\leq i\leq n+1\right),\\ {\hat{e}}_{i,2}=\left\langle -v_{i,2}^{+},+v_{i,2}^{-},0,0\right\rangle \;\left(1\leq i\leq n+1\right), \end{array}$$

and *E_L _*consists of

$$\begin{array}{c} e_{Li}=\left\langle +v_{i,1}^{-},-v_{n+1,2}^{+},0,0\right\rangle \;\left(1\leq i\leq n\right),\\ e_{Li}=\left\langle -v_{i,2}^{+},+v_{n+1,1}^{-},0,0\right\rangle \;\left(1\leq i\leq n\right). \end{array}$$

**Figure 9:**
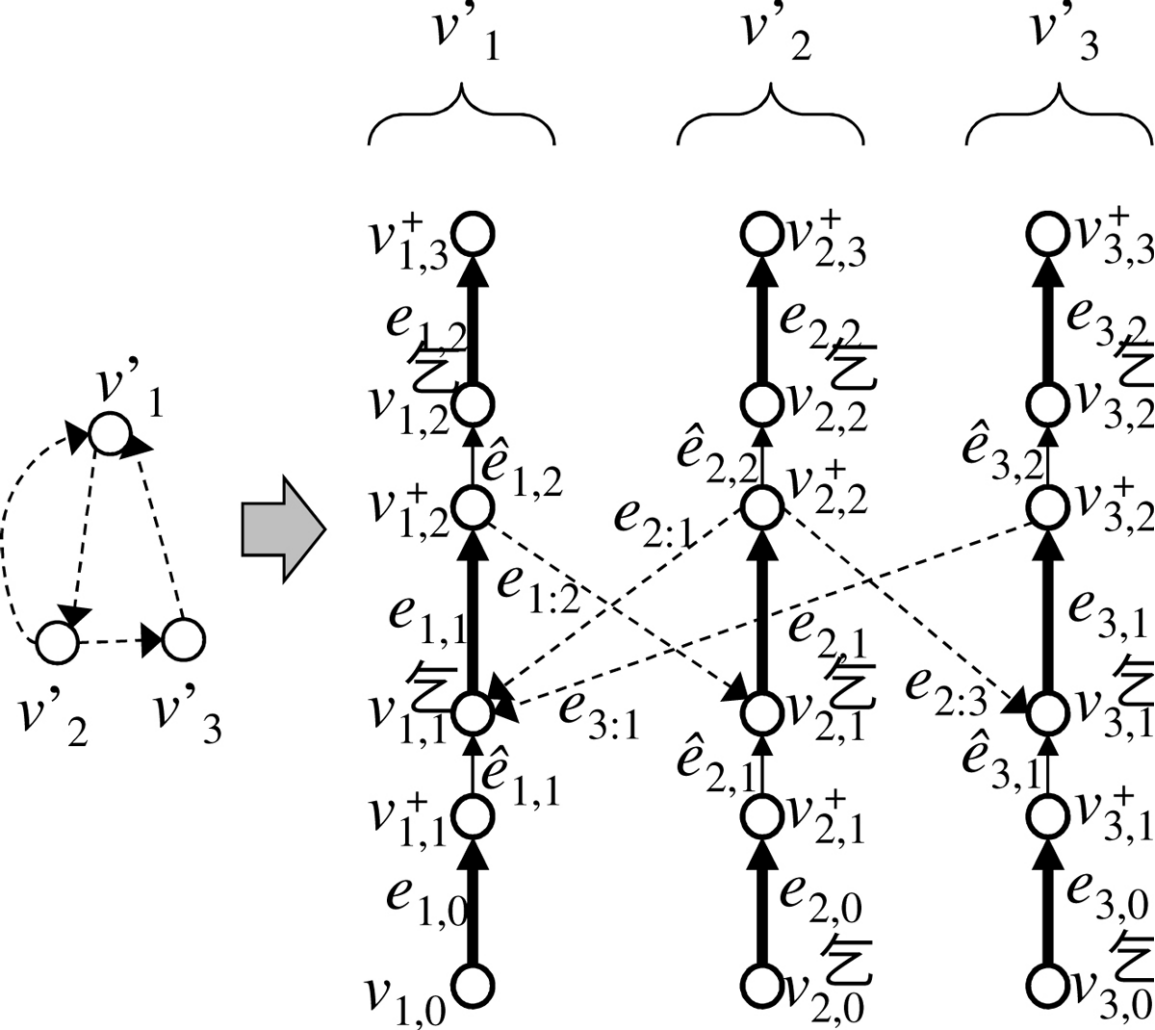
**An example of a chromosome graph for solving the partition problem (PARTITION)**. In this example, *n *= 4.

We set *λ_i _*= 10*S*_Σ _for any *i *≥ 1, *Q_N _*= *Q_T _*= 100*S*_Σ_, *n_N _*= *n*+2, and *n_T _*= 0. See Figure 9 for an example. In addition, we set *V_W _*to *V*_5 _∪ *V*_3_, and *E_W _*to *E *by making all edges in *E_L _*∪ *E_R _*required so that *G *satisfies WCC.

We show that PARTITION for *S *has a solution *S' *⊂ *S *if, and only if, there exists a solution *C *of ChrL such that *W*(*C*) = 0. First, suppose that PARTITION has a solution *S'*. Let *r*_*S' *_be a cycle generated by merging cycles $e_{n+1,1}e_{Li}e_{i,1}e_{Li}^{\prime }$ for *i *∈ *S'*. We define *r*_*S*-*S' *_in the same way. Consider a multi-set *C *= {*c*_1_,..., *c*_*n*+2_}, where *c_i _*∈ *C *is a chromosome on *G *such that

$$\begin{array}{c} c_{i}\;=\;e_{i,0}{\hat{e}}_{i,1}e_{i,1}{\hat{e}}_{i,2}e_{i,2}\;\left(1\leq i\leq n\right),\\ c_{n+1}=e_{n+1,0}{\hat{e}}_{n+1,1}r_{S^{\prime }}e_{n+1,1}{\hat{e}}_{n+1,2}e_{n+1,2},\\ c_{n+2}=e_{n+1,0}{\hat{e}}_{n+1,1}r_{S-S^{\prime }}e_{n+1,1}{\hat{e}}_{n+1,2}e_{n+1,2}. \end{array}$$

Then, *W*(*C*) = 0 because |*C*| = *n *+ 2, Tr(*C*) = 0, and *m*(*C*, *e*) = *n*(*e*) for *e *∈ *E_S_*. In addition, *C *visits all required edges. Furthermore, |*c_i_*| = 10Σ ≤ *λ_i _*for 1 ≤ *i *≤ *n *+ 2.

Conversely, suppose that ChrL for *G *has an optimal solution *C *that satisfies *W*(*C*) = 0. Because *W*(*C*) = 0, we obtain |*C*| = *n *+ 2, Tr(*C*) = 0, and *m*(*C*, *e*) = *n*(*e*) for *e *∈ *E*. Because ∑_*e*∈*E*_|*e*|*n*(*e*) = 10(*n *+ 2)*S*_Σ_, |*c*| = 10Σ for each *c *∈ *C*. Let *c*_*i *_be a chromosome that begins with *e*_*i*,0 _for 1 ≤ *i *≤ *n*. The other two chromosomes are denoted by *c*_*n*+1 _and *c*_*n*+2_. Then, *c*_1 _begins with $e_{1,0}{\hat{e}}_{1,1}e_{1,1}$. Suppose that *c*_1 _does not visit ${\hat{e}}_{1,2}e_{1,2}$. Then, there is a chromosome *c_i _*that visits ${\hat{e}}_{1,2}e_{1,2}$, whose previous edge has to be *e*_1,1 _in *c*_*i*_. Therefore, for some paths *p*_1 _and *p*_2_,

(7) $$\begin{array}{c} c_{1}=e_{1,0}{\hat{e}}_{1,1}e_{1,1}p_{1}\\ c_{i}=p_{2}e_{1,1}{\hat{e}}_{1,2}e_{1,2}. \end{array}$$

Because of (7), $\left|c_{1}|=|e_{1,0}|+|{\hat{e}}_{1,1}|+|e_{1,1}|+|p_{1}|=10S_{\Sigma }=|e_{1,0}|+|{\hat{e}}_{1,1}|+|e_{1,1}|+|{\hat{e}}_{1,2}|+|e_{1,2}\right|$. Therefore, $\left|p_{1}|=|{\hat{e}}_{1,2}|+|e_{1,2}\right|$. We modify *C *so that

$$\begin{array}{c} c_{1}=e_{1,0}{\hat{e}}_{1,1}{\hat{e}}_{1,2}e_{1,2,}\\ c_{i}=p_{2}e_{1,1}p_{1}. \end{array}$$

The modified *C *still satisfies the required conditions. After this modification is repeated for 2 ≤ *i *≤ *n *until no more modifications can be applied, *C *satisfies $c_{i}=e_{i,0}{\hat{e}}_{1,1}e_{i,1}{\hat{e}}_{i,2}e_{i,2}$ for 1 ≤ *i *≤ *n*. Another chromosome exists that visits *e*_*i*,1 _for each 1 ≤ *i *≤ *n*, which is one of *c*_*n*+1 _and *c*_*n*+2_. Let *S' *= {*i*|*m*(*c*_*n*+1_, *e*_*i*,1_) *>*0}. Then, ∑_*i*∈*S'*_*s*(*i*) = 10*S*_Σ _− (9*/*2+5)*S*_Σ _= 1*/*2*S*_Σ_. Therefore, *S' *is a solution of PARTITION.

## Competing interests

The authors declare that they have no competing interests.

## Authors' contributions

TY formulated the problem, proved the NP-completeness, developed a polynomial-time algorithm, and composed the manuscript. SM critically revised the manuscript.

## Declarations

TY would like to acknowledge financial support from the Human Genome Center, Institute of Medical Science, University of Tokyo.

This article has been published as part of *BMC Genomics *Volume 16 Supplement 2, 2015: Selected articles from the Thirteenth Asia Pacific Bioinformatics Conference (APBC 2015): Genomics. The full contents of the supplement are available online at http://www. biomedcentral. com/bmcgenomics/supplements/16/S2
