# Erratum to: inferring the global structure of chromosomes from structural variations

**DOI:** 10.1186/s12864-015-1338-2

**Published:** 2015-04-09

**Authors:** Tomohiro Yasuda, Satoru Miyano

**Affiliations:** The Human Genome Center, Institute of Medical Science, University of Tokyo, Shiroganedai, Minato-ku, Tokyo Japan; Department of Computer Science, University of Tokyo, Hongo, Bunkyo-ku, Tokyo Japan

## Corrections

After publication of [1] we became aware that author revisions had not been incorporated into the final published version. The following corrections should be made. A PDF version into which all corrections are incorporated is attached as Additional file 1.

### Formatting

Incorrectly formatted descriptions in [1] should be corrected as follows.

1. In the original publication, the images of Figures 1, 3, 5–9 are shuffled. In addition, images of Figures 1, 3, 4, 6–9 contain incorrectly encoded symbols. They should be replaced with Figures (1, 2, 3, 4, 5, 6, 7, 8, 9) presented in this article.

**Figure 1:**
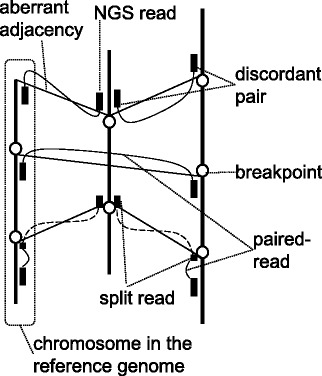
**Aberrant adjacencies of genomic regions.** Thick vertical lines represent chromosomes in the reference genome, circles represent breakpoints, small black boxes represent NGS reads, solid curved lines represent paired-reads, dashed curved lines represent split reads, and thin solid oblique lines represent aberrant adjacencies. Aberrant adjacencies are detected by using two types of NGS reads abnormally mapped to the reference genome: discordant pairs (three pairs from above), and split reads (two pairs from below).

**Figure 2:**
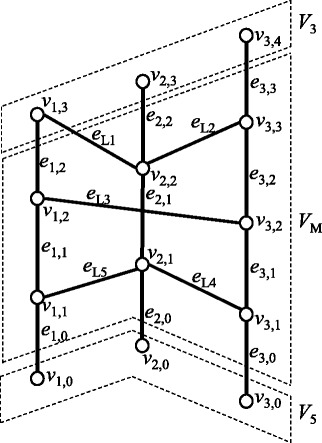
**The caption of this figure is omitted because it was correct in the original publication.**

**Figure 3:**
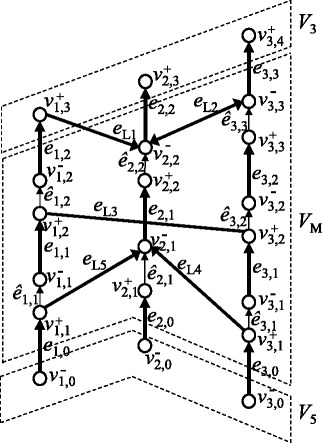
**An example of a chromosome graph.** Thin vertical edges represent edges in *E*_*R*_. Arrowheads represent the ‘ + ’-direction, whereas ends of edges without arrowheads represent ‘ − ’-direction.

**Figure 4:**
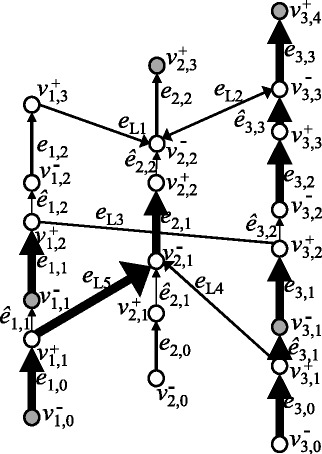
**An example of a chromosome graph that satisfies WCC.** Gray circles are vertices in *V*_*W*_ and thick arrows are edges in *E*_*W*_.

**Figure 5:**
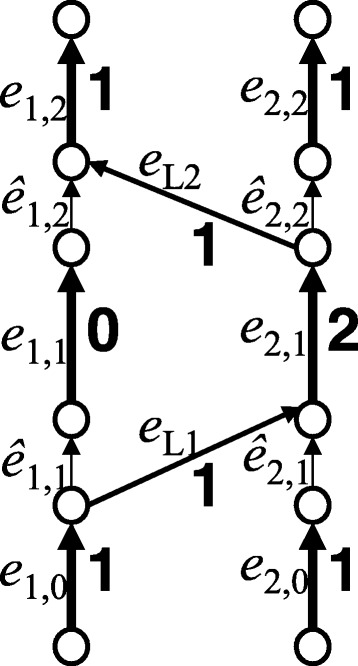
**An example of a chromosome graph that has more than one optimal solution.** Bold digits represent an optimal circulation on this graph. The chromosome graph in this figure has two optimal solutions {*e*_1,0_ *e*_*L*1_ *e*_2,1_ *e*_*L*2_ *e*_1,2_, *e*_2,0_ *ê*_2,1_ *e*_2,1_ *ê*_2,2_ *e*_2,2_} and {*e*_1,0_ *e*_*L*1_ *e*_2,1_ *ê*_2,2_ *e*_2,2_, *e*_2,0_ *ê*_2,1_ *e*_2,1_ *e*_*L*2_ *e*_1,2_}. Edges in *E*_*N*_∪*E*_*D*_ are omitted, and the flow on each edge in *E*_*D*_ has been subtracted from the flow of a corresponding edge in *E*_*S*_.

**Figure 6:**
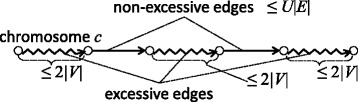
**An example of a chromosome that consists of non-excessive and excessive edges.** Straight arrows represent non-excessive edges, while jagged lines represent sequences of excessive edges.

**Figure 7:**
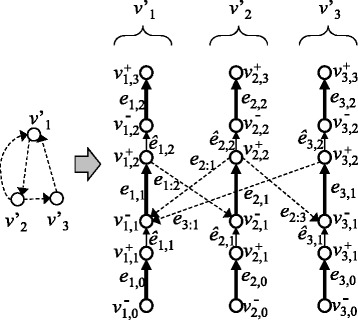
**An instance of ChrP for solving the Hamiltonian Cycle problem (HC).** In this graph, solid edges are constructed for each vertex in a graph *H* of HC, whereas dashed edges correspond to edges in *H*.

**Figure 8:**
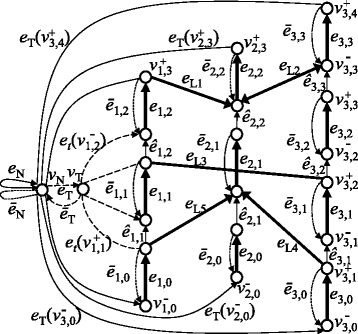
**An example of a circular chromosome graph.** The problem of optimizing multiple chromosomes is converted to the problem of finding a cycle on this graph. For simplicity, we omitted *e*_*t*_ (•), except for the leftmost chromosome in the reference genome.

**Figure 9:**
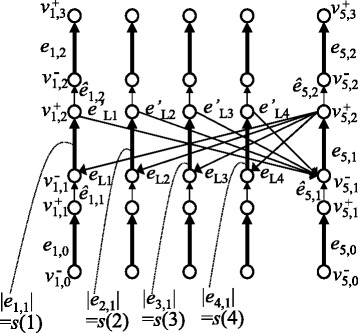
**An example of a chromosome graph for solving the partition problem (PARTITION).** In this example, *n* = 4.
2. All four occurrences of “Yasuda and Miyano Page *n* of 11” in the main text should be removed.
3. All three occurrences of “*O*(|*E*|_2_ log |*V* | log |*E*|)” should read “*O*(|*E*|^2^ log |*V* | log |*E*|)”
4. In the Results, both of two “CC($\tilde{G}$, *E*+)” should read “CC($\tilde{G}$, *E*_+_)”
5. In the Methods, a Q.E.D. symbol “☐(white box)” should be inserted at the end of the following lines:
  The line just before Lemma 2
  The line just before Lemma 3
  The second line from below before “Proof of Theorem 2”, ending with “a Hamiltonian cycle on *H*.”
  The line with the sentence “Therefore, *C* satisfies (6).” in subsection *Circular chromosome graph*
6. In the Background, “BCRABL” should read “BCR-ABL”
7. In the Methods, in the proof of *Lemma 1*, the expression “*c* = *p*_1_*e*_1_*p*_2_*e*_2_ . . . *e*_*t*_*cp*_*t*_*c*_+1_” should read “ $c={p}_1{e}_1{p}_2{e}_2.\ .\ .{e}_{t_c}{p}_{t_c+1}$”
8. In the same proof, just above *Lemma 2*, the expression “2|*V* |(*n*_*N*_+*n*_*T*_ )+(4|*V* |+1)P_*e*_ ∗ ∗ ∗_*E*_S *n*(*e*) ≤*U*(4|*V*|+1)(|*E*|+1)” should read “ $2\left|V\right|\left({n}_N + {n}_T\right) + \left(4\left|V\right| + 1\right){\displaystyle {\sum}_{e\in {E}_S}n(e)}\ \le U\left(4\left|V\right| + 1\right)\left(\left|E\right|+1\right)$”
9. In the Methods, in the proof of *Lemma 3*, the following expressions
  $$ {e_i}_{,1}=<-{v}_{i,1}^{-},+{v}_{i,2}^{\ddagger },1,1>\left(2\le\ i\le \left|V\hbox{'}\right|\right), $$
  $$ {e_i}_{,2} = <-{v}_{i,2}^{-},+{v}_{i,3}^{\ddagger },0,1>\left(2\le\ i\le \left|V\hbox{'}\right|\right). $$
  should read
  $$ {e_i}_{,1} = <-{v}_{i,1}^{-},+{v}_{i,2}^{+},1,1 > \left(2\ \le\ i\ \le\ \left|V\hbox{'}\right|\right), $$
  $$ {e_i}_{,2}=<-{v}_{i,2}^{-},+{v}_{i,3}^{+},0,1 > \left(2\ \le\ i\ \le\ \left|V\hbox{'}\right|\right). $$
10. In the Methods, in the proof of *Lemma 4*, in the paragraph that begins with “All of these steps”, the expression “*m*(*C*, *e*) = *f*(*e*) + *f*(*ē*)(∈*E*_*S*_).” should read “*m*(*C*, *e*) = *f*(*e*) + *f*(*ē*) (*e*∈*E*_*S*_).” (Insert a white space before “(*e*∈*E*_*S*_)”.)
11. In the same proof, just before “Therefore, *C* satisfies (6).”, the expression “*w*(*e*,*m*(*C*, *e*))=0(*e*∈*E*_*L*_∪*E*_*R*_).” should read “*w*(*e*,*m*(*C*, *e*))=0 (*e*∈*E*_*L*_∪*E*_*R*_).” (Insert a white space before “(*e*∈*E*_*L*_∪*E*_*R*_)”.)

### Inaccurate descriptions

The following items correct inaccurate descriptions in the original manuscript. We regret any inconvenience that they might have caused.

1. In the Results, in subsection *Formulation of the problem*, the phrase “its computational complexity was not analyzed” should read “its computational complexity was not intensively analyzed”
2. In the Results, in the first paragraph of subsection *Polynomial-time solvable variation*, both of two “*E*_*L*_∪*E*_*R*_” should read “*E*”
3. In the Results, in *Definition 2*, the phrase “if all *g*∈CC(G, *E*_*W*_) are good” should read “if all *g*∈CC(*G*, *E*_*W*_) are good and *n*(*e*) = 0 for *e*∈*E* − *E*_*W*_ ”
4. In the Results, in the paragraph just after *Definition 2*, the expression “*E*_*W*_ = {*e*∈*E*_*S*_ |*n*(*e*) ≥ 1}∪{*e*∈*E*_*L*_∪*E*_*R*_ |*e* is required}” should read “*E*_*W*_ = {*e*∈*E* |*e* is required}”
5. In the Results, the last sentence that begins with “Finally, if some” just before *Definition 3* should read as follows:
  “In addition, if some *g*∈CC(*G*, *E*_*W*_) that are not good still remain, edges *e* in *g* are forcibly removed from *E*_*W*_ by changing *e* not required and setting *n*(*e*) to 0. Finally, if *n*(*e*) > 0 for some *e*∈*E*− *E*_*W*_, *e* is changed to be required and added to *E*_*W*_ by confirming its existence, or *n*(*e*) is forcibly set to 0.”
6. In the Results, *Definition 3* should read “Let *G* = (*V*, *E* ) be a chromosome graph that satisfies WCC with respect to given *V*_*W*_ ⊂ V and *E*_*W*_ ⊂ *E*. Then, find a set *C* of chromosomes on *G* that minimizes *W*(C) when (3) is satisfied, each *v* ∈ *V*_*W*_ is at an end of some *c*∈*C*, and each *e*∈*E*_*W*_ appears in *C*.”
7. In the Methods, in the paragraph just above *Lemma 4*, the sentences “For *e*∈*E*_*S*_ ∪ {*e*_*N*_, *e*_*T*_ }, we set *l*(*e*) = *n*(*e*), *l*(*ē*) = 0, and *u*(*ē*) = *n*(*e*). For *e*∈*E*_*L*_∪*E*_*R*_, we set *l*(*e*) = 1.” should read “For *e*∈*E*_S_∪{*e*_*N*_, *e*_*T*_}, we set *l*(*e*) = *n*(*e*), *l*(*ē*) = 0, and *u*(*ē*) = *n*(*e*) if *e* is not required, whereas *l*(*e*) = max{*n*(*e*), 1}, *l*(*ē*) = 0, and *u*(*ē*) = max{*n*(*e*) − 1, 0} if *e* is required. We assume that *e*_*N*_ is required because *n*_*N*_ ≥1. We also assume that *e*_*T*_ is required if |*V*_*W*_| ≥ 1. For *e*∈*E*_*L*_∪*E*_*R*_, we set *l*(*e*) = 1 if *e* is required, or *l*(*e*) = 0 otherwise.”
8. In the Methods, in the paragraph just after *Lemma 4*, the description “or *n*(*e*) ≥ 1” should be removed.
9. In the Methods, in subsection *Proof of Theorem 3*, the phrase “by making all edges in *E*_*L*_∪*E*_*R*_ required” should read “by making all edges required”

## Additional file

Additional file 1:
**Corrected version.** A PDF version of [1] into which all corrections in this article are incorporated.
