# Supplementary material for: Erratum to: inferring the global structure of chromosomes from structural variations
Source: BMC Genomics. 2015 Apr 9;16(1):276. doi: 10.1186/s12864-015-1338-2 (PMC4391112; doi:10.1186/s12864-015-1338-2)
Supplement: Additional file 1: — Corrected version. A PDF version of [1] into which all corrections in this article are incorporated. [file 12864_2015_1338_MOESM1_ESM.pdf]

RESEARCH

Inferring the global structure of chromosomes from structural variations

Tomohiro Yasuda[1,2] and Satoru Miyano[1*]

From The Thirteenth Asia Pacific Bioinformatics Conference (APBC 2015)
HsinChu, Taiwan. 21-23 January 2015

Abstract

Background: Next generation sequencing (NGS) technologies have made it possible to exhaustively detect structural variations (SVs) in genomes. Although various methods for detecting SVs have been developed, the global structure of chromosomes, i.e., how segments in a reference genome are extracted and ordered in an unknown target genome, cannot be inferred by detecting only individual SVs.

Results: Here, we formulate the problem of inferring the global structure of chromosomes from SVs as an optimization problem on a bidirected graph. This problem takes into account the aberrant adjacencies of genomic regions, the copy numbers, and the number and length of chromosomes. Although the problem is NP-complete, we propose its polynomial-time solvable variation by restricting instances of the problem using a biologically meaningful condition, which we call the *weakly connected constraint*. We also explain how to obtain experimental data that satisfies the weakly connected constraint.

Conclusion: Our results establish a theoretical foundation for the development of practical computational tools that could be used to infer the global structure of chromosomes based on SVs. The computational complexity of the inference can be reduced by detecting the segments of the reference genome at the ends of the chromosomes of the target genome and also the segments that are known to exist in the target genome.

Background

Next-generation sequencing (NGS) technologies have drastically reduced the cost of genome sequencing [1]. As more genomic sequences have become available, it has become clear that genomes contain many *structural variations (SVs)*, which include large insertions, deletions, tandem duplications, and translocations. SVs have already been associated with diverse diseases [2]. For example, the fusion genes BCR-ABL and EML4-ALK play key roles in the development of cancer, and it is believed that other recurrent rearrangements remain to be discovered [3]. In cancer genomes, many SVs are occasionally concentrated in a small region of the genome [4, 5, 6]. It has been suggested that a single catastrophic mutational event, known as *chromothripsis* [6], causes these concentrations. A study of prostate cancer also uncovered a distinct type of complex rearrangement termed *chromoplexy* [7, 8], wherein rearrangements are unclustered but involve multiple chromosomes. Complex genomic rearrangements have even been observed in germline mutations, resulting in serious congenital diseases [9]. Because of their importance in functions of the genome, various methods have been developed for finding SVs [10, 11, 12, 13, 14, 15, 16]. When genomic rearrangements are complex, enumerating only individual SVs is insufficient for elucidating the *global structure of chromosomes*, i.e., how the segments in a reference genome are extracted and ordered in an unknown target genome. Here, the *reference genome* is known and is a pre-existing sequenced genome of the same organism, such as the GRCh38 build of the human genome [17].

In this study, we address the problem of inferring the global structure of chromosomes based on *SV data*, which refer to aberrant adjacencies of genomic regions and copy number variations (CNVs) in this study. By solving this problem, we can determine the order of the genomic regions in the target genome. This order affects the structure of proteins if the genomic regions

*Correspondence: miyano@hgc.jp
[1]The Human Genome Center, Institute of Medical Science, University of Tokyo, Shiroganedai, Minato-ku, Tokyo, JP
Full list of author information is available at the end of the article

contain coding regions, and regulation of genes if the genomic regions include promoters or enhancers. In addition, raw SV data could be corrected by inferring the global structure of chromosomes because an optimal global structure would ignore false positive detection of aberrant adjacencies or correct wrongly estimated copy numbers. The task of inferring chromosomes is formulated as an optimization problem on a graph, which we term as a *chromosome graph.* Our contributions are summarized as follows:

- To infer the global structure of chromosomes, we formulate a computational problem that takes into account the number and length of chromosomes, as well as aberrant adjacencies and CNVs caused by genomic rearrangements. By taking SV data as the input, relatively low-depth NGS sequencing can be used.
- We prove that the problem is NP-complete.
- We propose a biologically meaningful restriction that makes the problem solvable in polynomial time. We also show an algorithm that solves the restricted problem.

Oesper et al. [18] presented a pioneering work that aimed to infer the global structure of chromosomes from SV data. They formulated the *copy number and adjacency genome reconstruction problem.* Their formulation is based on graphs that they termed *interval-adjacency graphs.* These graphs are essentially the same as our chromosome graphs, except that we used bidirected graphs [19, 20] while they used alternating paths to exclude paths on the graph that do not correspond to chromosomes. They also implemented an efficient algorithm called *paired-end reconstruction of genome organization (PREGO)* that solved their problem and obtained promising results. Our work includes the following results that were not addressed by Oesper et al. First, we present a formulation that takes into account the number and length of chromosomes determined experimentally. Second, we prove that the problem is NP-complete. Finally, we propose a variation of the problem that can be solved in polynomial time.

Some methods can also be applied to analyze the global structure of genomes by using non-SV data. First, *de novo* sequence assembly aims at reconstructing target genomes from raw NGS sequences [19, 21, 22, 23, 24, 25]. It includes a step to order fragments of genomes obtained by assembling NGS sequences. The step is usually implemented as an optimization problem, involving searching for paths that cover all vertices or all edges corresponding to substrings of genome sequences [19, 21]. By contrast, we allow some vertices and edges to be ignored because some portions of the reference genome might not appear in the target genome. Second, reference-assisted assembly [26], also known as comparative assembly [27], aims at ordering segments of an unknown target genome by using known genomes of other organisms. By contrast, we order segments so that the chromosomes in the solution are most consistent with the SV data and the experimentally determined number and length of chromosomes. Finally, methods based on permutations of integers [28] compare two genomes represented by two sequences of integers corresponding to genes or markers in the genome. Instead of using such sequences, we exploit SV data.

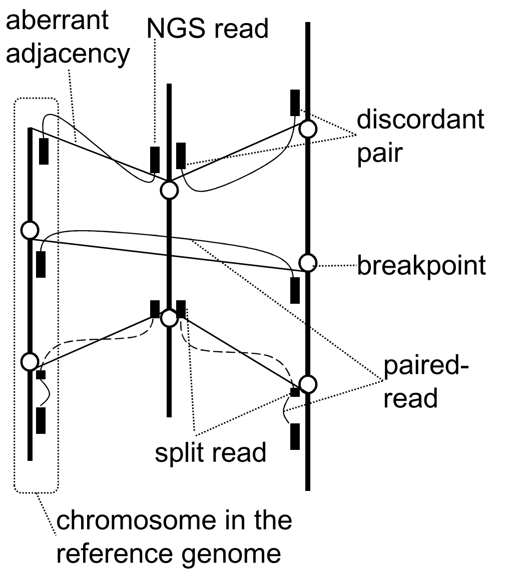

Figure 1 Aberrant adjacencies of genomic regions. Thick vertical lines represent chromosomes in the reference genome, circles represent breakpoints, small black boxes represent NGS reads, solid curved lines represent paired-reads, dashed curved lines represent split reads, and thin solid oblique lines represent aberrant adjacencies. Aberrant adjacencies are detected by using two types of NGS reads abnormally mapped to the reference genome: discordant pairs (three pairs from above), and split reads (two pairs from below).

The rest of this paper is organized as follows. First, we present types of experimental data from which we infer the global structure of chromosomes. Next, we give our formulation of the problem of inferring the global structure of chromosomes, and show that the problem is NP-complete. Then, we show a variation of the problem that is solvable in polynomial time. Finally, we discuss our results and state our conclusions.

Results

Experimental data

We assume the following experimental data as input.

Aberrant adjacencies
In the target genome, distant segments in the reference genome may be adjacent because of rearrangements (Figure 1). Such aberrant adjacencies are detected by using NGS technologies as follows. First, NGS technologies can generate read pairs that are a few hundred bases apart from each other in the target genome. If two reads of a pair are not mapped to the reference genome with the expected orientations and mapped distance, the pair is called a *discordant pair* and is likely to be caused by SVs [12, 13, 14]. Second, if the alignment of a read and reference genome is split into more than one portion, such a split read also indicates a rearrangement [16]. A *breakpoint* is a position at a boundary of a rearrangement. Here, we ignore small differences between the real breakpoints and their estimations.

Copy numbers
The number of occurrences of a subsequence in the reference genome may change because of rearrangements. This phenomenon results in *copy number variations (CNVs)*. Traditionally, CNVs have been analyzed by using DNA microarrays [11]. Several recent methods detect CNVs by finding changes in the depth of coverage of NGS sequences [4, 15]. Although tumor samples are usually a mixture of normal cells and various tumor cells, the copy numbers of a cancer cell can still be estimated by single-cell analysis [29]. In this paper, for the sake of conciseness, the boundaries of CNVs are also called *breakpoints*.

Number of chromosomes and truncations
Identifying chromosomes and finding aberrant chromosomes by microscopy is an important part of clinical diagnostics [30]. The number of chromosomes, denoted by n_N in this paper, is available after inspection. Throughout this paper, we assume that $n_N \geq 1$. In addition, we also take into account the number of chromosomal truncations, which we denote as n_T. Chromosomal truncations are detected as a decrease in copy numbers without aberrant adjacencies. We consider n_N and n_T to improve the inference of the global structure of chromosomes from SV data.

Chromosome length
The length of chromosomes can be estimated experimentally from flow karyotyping, and, approximately, from microscopic images [31]. Here, the estimated length is denoted by λ_i for $1 \leq i \leq N_L$, where $N_L(\geq n_N)$ is the maximum possible number of chromosomes.

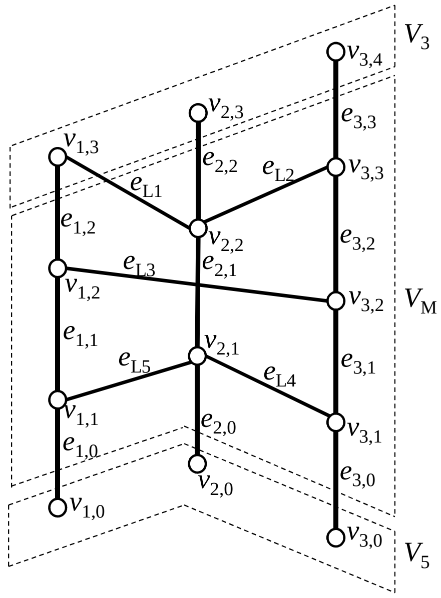

Figure 2 An example of a prototype chromosome graph. Thick vertical edges represent edges in E_S that correspond to segments in the reference genome, oblique edges represent edges in E_L that correspond to aberrant adjacencies. Vertices surrounded by dashed lines belong to V_5, V_M, and V_3, read from the bottom of the graph to top.

Problem definition
Any instance of our problem is modeled as a graph that we term a *chromosome graph*. The graph contains elements derived from the reference genome and experimental data. Each vertex corresponds to a location in the reference genome. In addition, each edge corresponds to either a segment in the reference genome, an adjacency of flanking segments in the reference genome, or an aberrant adjacency in the target genome caused by rearrangements.

We assume that the target genome is a set of chromosomes, each of which is a concatenation of segments in the reference genome. Each chromosome in the target genome is represented as a path on the graph, and these paths explain how segments in the reference genome are incorporated into the target genome. The goodness of the estimated target genome is measured by a cost function, and we search for an optimal set of chromosomes that minimizes this cost function.

We first define a graph that contains some of elements described above. Then, we extend the graph to a chromosome graph. Finally, we present the formal definition of the problem.

Prototype chromosome graph
We first construct an undirected graph called a *prototype chromosome graph*, $G = (V, E)$ (Figure 2). Let

N_C be the number of chromosomes of the reference genome and n_i be the number of breakpoints in the i-th chromosome of the reference genome. Then, V contains the following vertices.

- Vertices corresponding to breakpoints:

$$V_M = \{v_{i,j} | 1 \leq i \leq N_C, 1 \leq j \leq n_i\}.$$

- Vertices corresponding to the beginning of chromosomes in the reference genome:

$$V_5 = \{v_{i,0} | 1 \leq i \leq N_C\}.$$

- Vertices corresponding to the end of chromosomes in the reference genome:

$$V_3 = \{v_{i,n_i+1} | 1 \leq i \leq N_C\}.$$

Then, we define $V = V_5 \cup V_3 \cup V_M$.

Next, we define a set of edges, E. We make the following two types of edges.

- Edges corresponding to segments between two breakpoints that are next to each other in the reference genome. For each $1 \leq i \leq N_C$ and $0 \leq j \leq n_i$, we make an edge $e_{i,j} = (v_{i,j}, v_{i,j+1})$.
- Edges corresponding to aberrant adjacency of two segments in the reference genome. Let N_A be the number of detected aberrant adjacencies. For the k-th aberrant adjacency $(1 \leq k \leq N_A)$ that links positions corresponding to v_{i_1,j_1} and v_{i_2,j_2}, we make an edge $e_{Lk} = (v_{i_1,j_1}, v_{i_2,j_2})$.

Then, we define

$$E_S = \{e_{i,j} | 1 \leq i \leq N_C, 0 \leq j \leq n_i\},$$
$$E_L = \{e_{Lk} | 1 \leq k \leq N_A\},$$
$$E = E_S \cup E_L.$$

Chromosome graph

In a prototype chromosome graph, a path might visit two edges in E_L contiguously. Such a path does not correspond to a real chromosome. To exclude such a path we use a technique similar to that of Oesper et al. [18]. Although Oesper et al. [18] used alternating paths, their formulation can be represented by using a bidirected graph whose edges have directions at both ends [19, 32]. We directly define our graph by using a bidirected graph (Figure 3). Let $d(e, v) \in \{+, -\}$ be the direction of an edge e at a vertex v, and $-d(e, v)$ be the opposite direction of $d(e, v)$.

- Each vertex $v_{i,j} \in V_M$ is split into two vertices $v^+_{i,j}$ and $v^-_{i,j}$. The set V_M is redefined as

$$V_M = \{v^-_{i,j}, v^+_{i,j} | 1 \leq i \leq N_C, 1 \leq j \leq n_i\}.$$

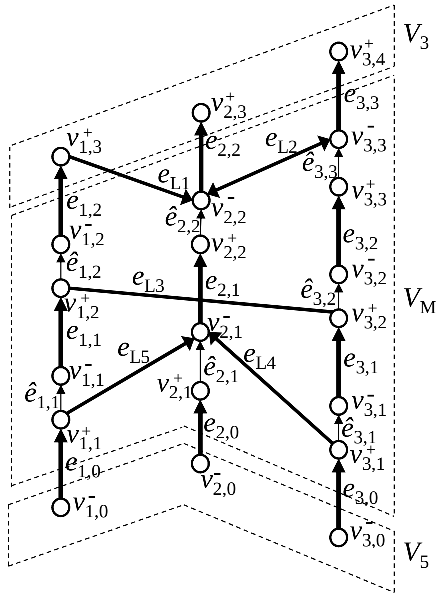

Figure 3 An example of a chromosome graph. Thin vertical edges represent edges in E_R. Arrowheads represent the '+'-direction, whereas ends of edges without arrowheads represent '−'-direction.

Vertices in V_5 and V_3 are renamed so that

$$V_5 = \{v^-_{i,0} | 1 \leq i \leq N_C\},$$
$$V_3 = \{v^+_{i,n_i+1} | 1 \leq i \leq N_C\}.$$

- An edge $e_{i,j} = (v_{i,j}, v_{i,j+1}) \in E_S$ is reconnected to $v^-_{i,j}$ and $v^+_{i,j+1}$. In addition, $d(e_{i,j}, v^-_{i,j}) = -$ and $d(e_{i,j}, v^+_{i,j+1}) = +$.
- Let $e \in E_L$ be an edge connected to $v_{i,j}$ in the prototype chromosome graph. If e corresponds to an aberrant adjacency involving the segment that stretches toward $v_{i,j+1}$, e is reconnected to $v^-_{i,j}$ and $d(e, v^-_{i,j})$ is set to '+'. Otherwise, e is reconnected to $v^+_{i,j}$ and $d(e, v^+_{i,j})$ is set to '−'.
- We add the following set of new edges:

$$E_R = \{\hat{e}_{i,j} = (v^+_{i,j}, v^-_{i,j}) | 1 \leq i \leq N_C, 1 \leq i \leq n_i\}.$$

Directions are set so that $d(\hat{e}_{i,j}, v^+_{i,j}) = -$ and $d(\hat{e}_{i,j}, v^-_{i,j}) = +$.

The modified graph represents a *chromosome graph.*

Paths and chromosomes

A *path* $c = v_1 e_1 v_2 e_2 v_3 \ldots e_l v_{l+1}$ on a chromosome graph G is an alternating sequence of vertices and edges, which has the following properties:

- The first and the last of c are vertices.

- Any subsequence of the form $e_k v_k e_{k+1}$ $(1 \leq k \leq l)$ means that $d(e_k, v_k) = -d(e_{k+1}, v_k)$.

A path c is said to *visit* an edge e if c contains e. Similarly, c is said to *visit* a vertex v if c contains v. When a path is written as a sequence of vertices and edges, for simplicity, we omit the notation of the vertices if they are clear. Let $C = \{c_1, c_2, \ldots, c_{|C|}\}$ be a multi-set of paths on G. We define C as a multi-set so that more than one identical path can exist. In addition, let $m(c, e)$ be the number of times c visits an edge e, and $m(C, e) = \sum_{c_i \in C} m(c_i, e)$. A *cycle* is a path whose first and last vertices are identical and the directions of the first and the last edges at the vertex are opposite. A *chromosome* on G is a path whose first and last edges are both in E_S.

Copy numbers and lengths

Two integers are assigned to each $e \in E$. First, $n(e)$ for $e \in E_S$ represents an experimentally estimated copy number of the corresponding segment in the reference genome. Second, $|e|$ for $e \in E_S$ represents the length of the corresponding segment in the reference genome. For $e \in E_L \cup E_R$, we set $n(e)$ and $|e|$ to 0. The length of a path c is defined as $|c| = \sum_{e \in E} |e| m(c, e)$. To simply describe all properties of e together, we use the following notation:

$$e = \langle d(e, v_1)v_1, d(e, v_2)v_2, n(e), |e| \rangle.$$

Upper bound on parameters

Campbell et al. [4] presented examples of amplified regions in cancer cells. The copy numbers were less than 100 in these regions. Therefore, we assume that the copy numbers are in at most hundreds. We also assume that short repeat elements are masked in advance in order to exclude segments that appear spuriously. Based on the details given above, we assume that n_N, n_T, and $n(e)$ for $e \in E_S$ are all less than a fixed constant U. The value of U does not have to be determined because U is only used in the analysis of computational complexity.

Formulation of the problem

To find an optimal set of chromosomes, we define an optimization problem over a chromosome graph. We define a cost function to be used as a target function of the optimization problem. This function imposes costs on the number of chromosomes, the number of chromosomal truncations, and the number of visits to edges, penalizing for deviations from those that are experimentally expected.

Let $C = \{c_1, c_2, \ldots, c_{|C|}\}$ be a multi-set of chromosomes on G, and $w_N(C)$ be the cost of the difference between n_N and $|C|$. Also let $\mathrm{Tr}(C)$ be the number of ends of chromosomes in V_M, and $w_T(C)$ be the cost of the difference between n_T and $\mathrm{Tr}(C)$. In addition, $w(e, x)$ for $e \in E_S$ is defined as the cost when e is visited x-times. For $e \in E_L \cup E_R$, $w(e, x)$ is set to 0.

We assume that $w_N(C)$, $w_T(C)$, and $w(e, x)$ for $e \in E_S$ monotonically increase as $||C| - n_N|$, $|\mathrm{Tr}(C) - n_N|$, and $|x - n(e)|$ increase, respectively. Then, we define the cost function $W(C)$ as follows:

$$W(C) = w_N(C) + w_T(C) + \sum_{e \in E} w\left(e, m(C, e)\right). \quad (1)$$

We assume that each term is 0 if and only if

$$\left.\begin{array}{rcl} |C| & = & n_N, \\ \mathrm{Tr}(C) & = & n_T, \\ m(C, e) & = & n(e) \text{ for } e \in E_S. \end{array}\right\} \quad (2)$$

With these notations, we formulate the problem of inferring the global structure of chromosomes as follows:

Definition 1 (Chromosome problem (ChrP)) *Suppose that we are given a chromosome graph $G = (V, E)$, a cost function $W(C)$, and parameters λ_i $(1 \leq i \leq N_L)$, where N_L is the maximum possible number of chromosomes. Then, find a multi-set of chromosomes C on G that minimizes $W(C)$ under the constraint that $|c_i| \leq \lambda_i$ for $c_i \in C$.*

Although a similar problem was proposed previously [18], its computational complexity was not intensively analyzed.

Theorem 1 *ChrP is NP-complete.*

In the Methods section, we prove Theorem 1.

Polynomial-time solvable variation

We propose a variation of ChrP that is solvable in polynomial time. For $e \in E$, it is highly likely that $m(C, e) \geq 1$ if e is supported by a large number of paired-reads. Therefore, it is worth considering a variation in which some edges in E must appear in the target genome. We refer to the edges as *required edges.* In addition, because chromosomal truncations can be detected, it is also worth considering a variation in which we know where the ends of the chromosomes of the target genome exist in the reference genome. Because the definition of $W(C)$ is abstract, we focus on a cost function such that

$$\left.\begin{array}{rcl} w_N(C) & = & Q_N||C| - n_N|, \\ w_T(C) & = & Q_T|\mathrm{Tr}(C) - n_T|, \\ w(e, x) & = & |e||x - n(e)|, \end{array}\right\} \quad (3)$$

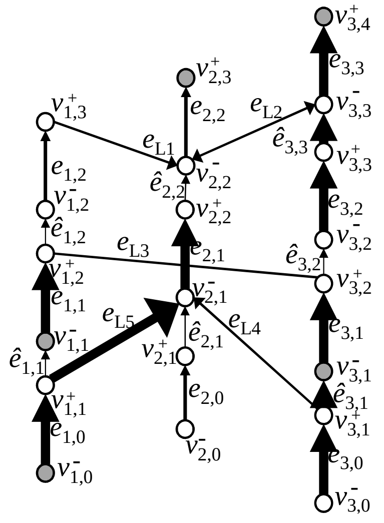

Figure 4 An example of a chromosome graph that satisfies WCC. Gray circles are vertices in V_W and thick arrows are edges in E_W.

where Q_N and Q_T are constants given as parameters. The values of Q_N and Q_T are tuned in advance so that known global structures of genomes are well reconstructed.

Weakly connected constraint

Let $G = (V, E)$ be a general bidirected graph. A subgraph g of G is a *weakly connected component* if g is a connected component when all directions are removed [33]. In addition, g is *maximal* if g is not a subgraph of a larger weakly connected component. For a subset E' of E, we define $\mathrm{CC}(G, E')$ as a set of maximal weakly connected components of a graph induced from G by removing the edges not in E'.

Definition 2 (Weakly connected constraint (WCC)) *Let $G = (V, E)$ be a chromosome graph. Also let V_W and E_W be subsets of V and E, respectively. Each $g \in CC(G, E_W)$ is* good *if g contains at least one vertex in V_W. Then, G satisfies the* weakly connected constraint (WCC) *if all $g \in CC(G, E_W)$ are good and $n(e) = 0$ for $e \in E - E_W$.*

We use WCC by setting V_W to a set of vertices that correspond to ends of chromosomes in the target genome, and $E_W = \{e \in E | e \text{ is required}\}$. See Figure 4 for an example. An instance that satisfies WCC can be obtained as follows. First, V_W is obtained by finding the positions of chromosomal truncations, as well as the ends of the chromosomes of the reference genome that remain in the target genome. Because a chromosome that does not include detected ends can be in a solution, V_W does not need to contain all ends of chromosomes in the target genome. We assume that $n_T \geq |V_W|$. Next, if $g \in \mathrm{CC}(G, E_W)$ is not good, edges $e \in E$ on some path connecting g and good $g' \in \mathrm{CC}(G, E_W)$ are added to E_W. To do this, if possible, we experimentally confirm that $n(e) \geq 1$ if $e \in E_S$ or that e is required if $e \in E_L \cup E_R$. In addition, if some $g \in \mathrm{CC}(G, E_W)$ that are not good still remain, edges e in g are forcibly removed from E_W by changing e not required and setting $n(e)$ to 0. Finally, if $n(e) > 0$ for some $e \in E - E_W$, e is changed to be required and added to E_W by confirming its existence, or $n(e)$ is forcibly set to 0.

Definition 3 (Chromosome problem with WCC (ChrW)) *Let $G = (V, E)$ be a chromosome graph that satisfies WCC with respect to given $V_W \subset V$ and $E_W \subset E$. Then, find a set C of chromosomes on G that minimizes $W(C)$ when (3) is satisfied, each $v \in V_W$ is at an end of some $c \in C$, and each $e \in E_W$ appears in C.*

Theorem 2 *The problem ChrW can be solved in $O(|E|^2 \log |V| \log |E|)$ time.*

See the Methods section for the algorithm that solves ChrW.

Restriction on the length of chromosomes

In ChrW, we removed restrictions on the length of chromosomes. This relaxation is necessary to make the problem solvable in polynomial time.

Definition 4 (ChrW with restriction on length (ChrL)) *ChrW with restriction on length (ChrL) is the same problem as ChrW, except that the length of each chromosome c_i is bounded by a parameter λ_i ($1 \leq i \leq N_L$), where N_L is the maximum possible number of chromosomes.*

Theorem 3 *The problem ChrL is NP-complete.*

See the Methods section for proof that problem ChrL is NP-complete.

Discussion

Handling practical situations

Solutions to the chromosome problems are affected by errors in given SV data. However, some errors can be mitigated as follows. First, a false positive aberrant adjacency may be correctly ignored in the optimal solution because a set of chromosomes that uses such an adjacency is expected to have a larger cost than those ignoring the adjacency. Second, the effects of a missing

aberrant adjacency may be limited to segments including its ends because a chromosome that contains the missing adjacency may be recognized as two split chromosomes. Finally, there is a chance that incorrect copy numbers will be corrected if they are inconsistent with other SVs.

In addition to segments in the reference genome, our method can handle newly inserted fragments not in the reference genome. Such a fragment is incorporated into a chromosome graph as a new chromosome. In particular, an edge e, where $|e|$ is equal to the length of the fragment, is added to E_S, and edges that connect vertices in a chromosome graph to e are added to E_L. If any breakpoints are contained within the new fragment, vertices and edges are added to V_M and E_R, respectively. If a breakpoint corresponds to any aberrant adjacency, edges are also added to E_L.

If a gene duplication has occurred in the target genome, it causes an increased copy number and aberrant adjacencies flanking the gene. If it is a tandem duplication, an aberrant adjacency connecting the upstream and downstream regions of the gene should exist. If these SVs exist in given SV data, any solution to our problem has to take into account gene duplication.

Limitations

A mixture of many cells cannot be handled because it is difficult to correctly estimate copy numbers. However, our method may generate meaningful results for data obtained from multiple cells if the sum of copy numbers is correctly estimated. In this case, the solution is a mixture of chromosomes of all cells in the sample, although some of the chromosomes might be fused.

Note that many optimal solutions may exist depending on how an optimal circulation is converted into chromosomes. (Figure 5). Choosing the right solution requires additional information such as the mate-pairs of long genomic fragments, or the result of experiments involving such techniques as fluorescence *in situ* hybridization (FISH) that indicate whether or not distant genomic regions are in the same chromosome.

Toward implementation

For implementation, we require an algorithm that can calculate an optimal circulation on the bidirected graph. It would be difficult to implement Gabow's algorithm because no efficient implementation is currently known. Another option would be to use Medvedev's algorithm [19]. Any solver for general integer programming could also be used, as demonstrated by Oesper et al. [18], although the computational time bound is not guaranteed.

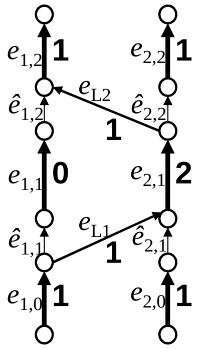

Figure 5 An example of a chromosome graph that has more than one optimal solution. Bold digits represent an optimal circulation on this graph. The chromosome graph in this figure has two optimal solutions $\{e_{1,0}e_{L1}e_{2,1}e_{L2}e_{1,2},\ e_{2,0}\hat{e}_{2,1}e_{2,1}\hat{e}_{2,2}e_{2,2}\}$ and $\{e_{1,0}e_{L1}e_{2,1}\hat{e}_{2,2}e_{2,2},\ e_{2,0}\hat{e}_{2,1}e_{2,1}e_{L2}e_{1,2}\}$. Edges in $E_N \cup E_D$ are omitted, and the flow on each edge in E_D has been subtracted from the flow of a corresponding edge in E_S.

Conclusions

Continuing technological innovations in DNA sequencing will, in future, allow the prediction of an enormous number of SVs. However, detecting only individual SVs cannot reveal the global structure of chromosomes. Here, we formulated the problem of inferring chromosomes from the aberrant adjacencies of genomic regions, copy number variations (CNVs), and the number and length of chromosomes. The problem, which we term as the *chromosome problem (ChrP)*, was proved to be NP-complete. However, if an instance of ChrP satisfies a constraint, which we call a *weakly connected constraint (WCC)*, and if the length of chromosomes is ignored, the problem can be solved in $O(|E|^2 \log |V| \log |E|)$ time.

This work provides a theoretical basis for the development of practical computational tools that are emerging for use in analysis of the global structure of chromosomes based on SVs.

Methods

In this section, we show how we proved the theorems stated in the Results section.

Proof of Theorem 1

We first present an upper bound on the size of an optimal solution of ChrP to show that ChrP is in NP. Then, we prove that ChrP is NP-hard.

Lemma 1 *Let $G = (V, E)$ be a chromosome graph. Also, let C be a multi-set of chromosomes on G that minimizes $W(C)$ such that $|c_i| \le \lambda_i$ for $c_i \in C$. Then, C has at most $U(4|V| + 1)(|E| + 1)$ edges.*

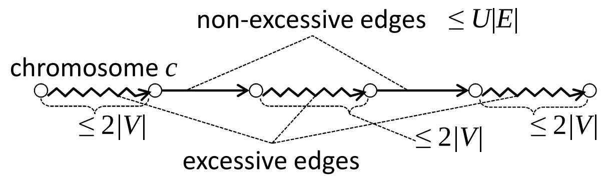

Figure 6 An example of a chromosome that consists of non-excessive and excessive edges. Straight arrows represent non-excessive edges, while jagged lines represent sequences of excessive edges.

Proof Let $c \in C$ be a chromosome in C. We define an edge e in c as *non-excessive* if $e \in E_S$ and $m(C, e) \leq n(e)$, and *excessive* otherwise. Let t_c be the number of non-excessive edges visited by c. If $t_c > 0$, c can be written as $c = p_1 e_1 p_2 e_2 \ldots e_{t_c} p_{t_c+1}$, where e_k $(1 \leq k \leq t_c)$ is a non-excessive edge and p_k $(1 \leq k \leq t_c+1)$ is a possibly empty path that contains only excessive edges (Figure 6). If p_k contains a cycle as its subpath, the cycle can be removed to decrease $W(C)$, a contradiction. Accordingly, p_k does not contain a cycle. This implies that p_k visits at most $2|V|$ vertices and, thus, $2|V|$ edges. Therefore, at most, $4|V|$ excessive edges are visited for each non-excessive edge. Note that a non-excessive edge e can be visited, at most, $n(e)$-times. Therefore, $\sum_{c \in C} t_c \leq \sum_{e \in E_S} n(e)$.

Chromosomes such that $t_c = 0$ can exist only if they contribute to the decrease of the first or the second term of $W(C)$ defined by (1). Accordingly, the number of such chromosomes is, at most, $n_N + n_T$. In addition, a chromosome c, such that $t_c = 0$, does not contain any cycles because such a cycle can be removed to decrease $W(C)$. Therefore, at most, c visits $2|V|$ vertices and, thus, $2|V|$ edges.

Consequently, C contains, at most, $2|V|(n_N + n_T) + (4|V|+1) \sum_{e \in E_S} n(e) \leq U(4|V|+1)(|E|+1)$ edges. □

Lemma 2 *The problem ChrP is in NP.*

Proof Once an optimal solution C is given, whether or not $W(C)$ is greater than a given constant can be determined in $O(|V||E|)$ time by Lemma 1. □

Lemma 3 *The problem ChrP is NP-hard.*

Proof The *Hamiltonian Cycle problem (HC)* is a problem of finding a cycle that visits each vertex of a graph exactly once, and is a well-known NP-complete problem [34]. Here, we reduce HC to ChrP. Consider HC on a directed graph $H = (V', E')$, where $V' = \{v'_1, v'_2, \ldots, v'_{|V'|}\}$ is a set of vertices and E'

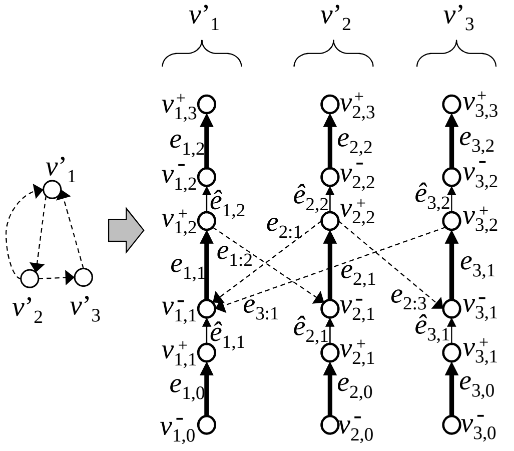

Figure 7 An instance of ChrP for solving the Hamiltonian Cycle problem (HC). In this graph, solid edges are constructed for each vertex in a graph H of HC, whereas dashed edges correspond to edges in H.

is a set of edges. We construct a chromosome graph $G = (V, E)$ from H (Figure 7), where

$$V = \bigcup_{1 \leq i \leq |V'|} \{v^-_{i,0}, v^+_{i,1}, v^-_{i,1}, v^+_{i,2}, v^-_{i,2}, v^+_{i,3}\}$$

is a set of vertices, and $E = E_S \cup E_L \cup E_R$ is a set of edges. Here, E_S consists of

$$\begin{aligned}
e_{1,0} &= \langle -v^-_{1,0}, +v^+_{1,1}, 1, 1 \rangle, \\
e_{1,1} &= \langle -v^-_{1,1}, +v^+_{1,2}, 2, 1 \rangle, \\
e_{1,2} &= \langle -v^-_{1,2}, +v^+_{1,3}, 1, 1 \rangle, \\
e_{i,0} &= \langle -v^-_{i,0}, +v^+_{i,1}, 0, 1 \rangle \quad (2 \leq i \leq |V'|), \\
e_{i,1} &= \langle -v^-_{i,1}, +v^+_{i,2}, 1, 1 \rangle \quad (2 \leq i \leq |V'|), \\
e_{i,2} &= \langle -v^-_{i,2}, +v^+_{i,3}, 0, 1 \rangle \quad (2 \leq i \leq |V'|).
\end{aligned}$$

E_R consists of

$$\begin{aligned}
\hat{e}_{i,1} &= \langle -v^+_{i,1}, +v^-_{i,1}, 0, 0 \rangle \quad (1 \leq i \leq |V'|), \\
\hat{e}_{i,2} &= \langle -v^+_{i,2}, +v^-_{i,2}, 0, 0 \rangle \quad (1 \leq i \leq |V'|).
\end{aligned}$$

E_L consists of

$$e_{i':i} = \langle -v^+_{i',2}, +v^-_{i,1}, 0, 0 \rangle \quad ((v'_{i'}, v'_i) \in E').$$

In addition, we set $n_N = 1$, $n_T = 0$, and $\lambda_i = |V'| + 3$ for any i. Then, we prove that H has a Hamiltonian cycle if, and only if, ChrP on G has a solution C such that $W(C) = 0$. Suppose that h is a Hamiltonian cycle on H. Let c be a chromosome that begins with $e_{1,0}\hat{e}_{1,1}e_{1,1}$ and then visits $e_{i':i}e_{i,1}$ in the order that edges $(v_{i'}, v_i)$ appear in h from $i' = 1$, and finally ends with $e_{1,1}\hat{e}_{1,2}e_{1,2}$. Then, a set of a single chromosome $C = \{c\}$ satisfies $W(C) = 0$ and $|c| = |V'| + 3 \leq \lambda_1$.

Conversely, let C be a solution of ChrP that satisfies $W(C) = 0$. Because (2) holds, $|C| = 1$, $\mathrm{Tr}(C) = 0$, and $m(C, e) = n(e)$. Let c be the only chromosome in C. Because $n(e_{1,1}) = 2$ and $n(e_{i,1}) = 1$ for $2 \le i \le |V'|$, a path that visits vertices $v'_i \in V'$ in the order that $e_{i,1}$ appears in c is a Hamiltonian cycle on H. □

Theorem 1 directly follows Lemma 2 and 3.

Proof of Theorem 2

Circulation on a bidirected graph

Let $G = (V, E)$ be a bidirected graph, and $a_{v,e}$ for $v \in V$ and $e \in E$ be an integer such that

$$a_{v,e} = \begin{cases} 2 & \text{if } e \text{ has two '+'-ends at } v, \\ 1 & \text{if } e \text{ has only one '+'-end at } v, \\ -1 & \text{if } e \text{ has only one '}-\text{'-end at } v, \\ -2 & \text{if } e \text{ has two '}-\text{'-ends at } v, \\ 0 & \text{if } e \text{ is not connected to } v. \end{cases}$$

Also let b_v be an integer defined for each $v \in V$, Z be the set of non-negative integers, and $l(e)$ and $u(e)$ be two non-negative integers assigned to each edge $e \in E$ called a *lower bound* and an *upper bound*, respectively. Unless otherwise specified, in this study $l(e) = 0$ and $u(e) = \infty$.

Definition 5 *A* bidirected flow (biflow) *[19, 20] is a mapping $f : E \to Z$ such that*

$$l(e) \le f(e) \le u(e) \qquad \text{for each } e \in E, \tag{4}$$

$$\sum_{e \in E} a_{v,e} f(e) = b_v \qquad \text{for each } v \in V. \tag{5}$$

The cost of f is defined as $W(f) = \sum_{e \in E} w(f, e)$, where $w(f, e)$ is a cost of f on $e \in E$. A circulation *is a biflow such that $b_v = 0$ for any $v \in V$.*

Circular chromosome graph

Definition 6 (Circular chromosome graph) *Let $G = (V, E)$ be a chromosome graph, and let v_N and v_T be new vertices. In addition, let E_N be a set of the following edges: for $1 \le i \le N_C$,*

$$\begin{aligned} e_t(v^-_{i,0}) &= \langle -v_N, +v^-_{i,0}, 0, 0 \rangle, \\ e_t(v^+_{i,n_i}) &= \langle -v_N, -v^+_{i,n_i}, 0, 0 \rangle, \\ e_t(v^+_{i,j}) &= \langle -v_T, -v^+_{i,j}, 0, 0 \rangle \quad (1 \le j \le n_i), \\ e_t(v^-_{i,j}) &= \langle -v_T, +v^-_{i,j}, 0, 0 \rangle \quad (1 \le j \le n_i), \end{aligned}$$

and

$$\begin{aligned} e_T &= \langle -v_N, +v_T, n_T, Q_T \rangle, \\ e_N &= \langle +v_N, +v_N, n_N, Q_N \rangle. \end{aligned}$$

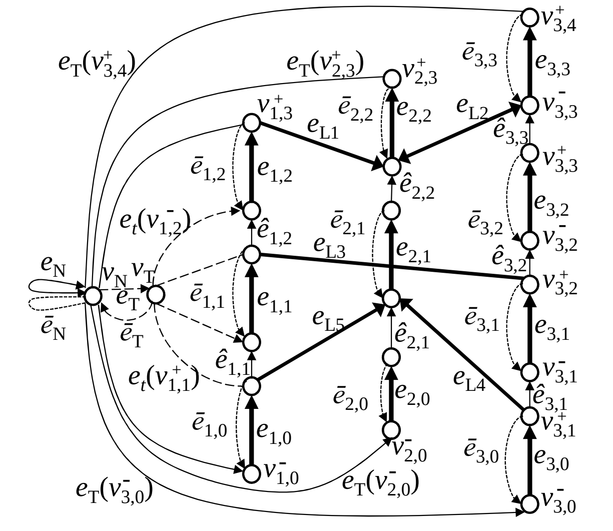

Figure 8 An example of a circular chromosome graph. The problem of optimizing multiple chromosomes is converted to the problem of finding a cycle on this graph. For simplicity, we omitted $e_t(\cdot)$, except for the leftmost chromosome in the reference genome.

Also, let E_D be a set of the following edges for $e \in E_S \cup \{e_N, e_T\}$:

$$\bar{e} = \langle -d(e, v_{i_1,j_1}) v_{i_1,j_1}, -d(e, v_{i_2,j_2}) v_{i_2,j_2}, 0, |e| \rangle,$$

where v_{i_1,j_1} and v_{i_2,j_2} are vertices at the ends of e. The graph $\tilde{G} = (V \cup \{v_N, v_T\}, E \cup E_N \cup E_D)$ is called a circular chromosome graph.

See Figure 8 for an example. Let $n(e_N) = n_N$ and $n(e_T) = n_T$. For $e \in E_S \cup \{e_N, e_T\}$, we set $l(e) = n(e)$, $l(\bar{e}) = 0$, and $u(\bar{e}) = n(e)$ if e is not required, whereas $l(e) = \max\{n(e), 1\}$, $l(\bar{e}) = 0$, and $u(\bar{e}) = \max\{n(e) - 1, 0\}$ if e is required. We assume that e_N is required because $n_N \ge 1$. We also assume that e_T is required if $|V_W| \ge 1$. For $e \in E_L \cup E_R$, we set $l(e) = 1$ if e is required, or $l(e) = 0$ otherwise. We also set $l(e_t(v))$ to 1 for $v \in V_W$ because these edges have to be visited in the solution.

Lemma 4 *Let $w(f, e) = |e| f(e)$ and $W_0 = Q_N n_N + Q_T n_T + \sum_{e \in E} |e| n(e)$. For any multi-set C of chromosomes on G, there is a circulation f on $\tilde{G}$ such that*

$$W(f) = W(C) + W_0. \tag{6}$$

Conversely, for any circulation f on $\tilde{G}$ that minimizes $W(f)$, there is a multi-set C of chromosomes on G that satisfies (6). In addition, C can be calculated in $O(\sum_{e \in E \cup E_N \cup E_D} f(e))$ time.

Let $E_+ = \{e \in E \cup E_N \cup E_D | l(e) \geq 1\}$. Note that $\mathrm{CC}(\tilde{G}, E_+)$ has only one weakly connected component because of WCC.

Proof First, we show that for any multi-set C of chromosomes on G, there exists a circulation f on $\tilde{G}$ that satisfies (6). Let $\mathrm{End}(v)$ be the number of chromosomes that begin or end with v. Consider the following f:

$$\begin{aligned} f(e) &= \max\{n(e), m(C,e)\} && (e \in E_S), \\ f(\bar{e}) &= \max\{0, n(e) - m(C,e)\} && (e \in E_S), \\ f(e) &= m(C,e) && (e \in E_L \cup E_R), \\ f(e_t(v)) &= \mathrm{End}(v) && (v \in V), \\ f(e_N) &= \max\{n_N, |C|\}, \\ f(\bar{e}_N) &= \max\{0, n_N - |C|\}, \\ f(e_T) &= \max\{n_T, \mathrm{Tr}(C)\}, \\ f(\bar{e}_T) &= \max\{0, n_T - \mathrm{Tr}(C)\}. \end{aligned}$$

Then, f is a circulation on $\tilde{G}$ because f satisfies (4) and (5). Thus, we observe that

$$w(e, m(C,e)) = |e|f(e) + |e|f(\bar{e}) - |e|n(e),$$

for $e \in E_S$, and

$$\begin{aligned} w_N(C) &= |e_N|f(e_N) + |e_N|f(\bar{e}_N) - Q_N n_N, \\ w_T(C) &= |e_T|f(e_T) + |e_T|f(\bar{e}_T) - Q_T n_T. \end{aligned}$$

Therefore, because $|e| = 0$ for $e \in E_L \cup E_R \cup \{e_t(v) | v \in V\}$ and $w(f,e) = |e|f(e)$, f satisfies (6).

Conversely, let f be a circulation on $\tilde{G}$ that minimizes $W(f)$. We show how to construct a multi-set C of chromosomes on G that satisfies (6).

First, for $e \in E_S \cup \{e_N, e_T\}$, we subtract $f(\bar{e})$ from $f(e)$, and also set $f(\bar{e})$ to 0.

Second, we construct a set R of cycles such that $m(R,e) = f(e)$ for any edge e in $\tilde{G}$. For directed graphs, the *flow decomposition theorem* [35] ensures that such R can be obtained in $O(\sum_{e \in E \cup E_N \cup E_D} f(e))$ time. This is also true for bidirected graphs.

Third, we merge cycles in R. Whenever a vertex is shared by two cycles in R, they are merged into a single cycle. Because of WCC, $\mathrm{CC}(\tilde{G}, E_+)$ consists of only one weakly connected component. This implies that all cycles that contain edges in E_+ can be merged into a single cycle. Note that any $r \in R$ contains at least one edge in E_+, because otherwise r can be removed to decrease $W(f)$. Therefore, all cycles in R can be merged into a single cycle $\tilde{r}$.

Finally, let C be a multi-set of paths generated by removal of v_N, v_T, and edges in E_N from $\tilde{r}$. Because $c \in C$ is connected to edges in E_N in $\tilde{r}$, the first and last edge of c is in E_S due to the directions of these edges. Accordingly, c is a chromosome. Therefore, C is a multi-set of chromosomes on G.

All of these steps can be completed in $O(\sum_{e \in E \cup E_N \cup E_D} f(e))$ time. In addition, we observe that the following equations hold:

$$\begin{aligned} |C| &= f(e_N) - f(\bar{e}_N), \\ \mathrm{Tr}(C) &= f(e_T) - f(\bar{e}_T), \\ m(C,e) &= f(e) + f(\bar{e}) \quad (e \in E_S). \end{aligned}$$

Accordingly, $w(e, m(C,e)) = w(f,e) + w(f,\bar{e}) + |e|n(e)$ for $e \in E_S$, and

$$\begin{aligned} w_N(C) &= w(f, e_N) + w(f, \bar{e}_N) + Q_N n_N, \\ w_T(C) &= w(f, e_T) + w(f, \bar{e}_T) + Q_T n_T, \\ w(e, m(C,e)) &= 0 \quad (e \in E_L \cup E_R). \end{aligned}$$

Therefore, C satisfies (6). □

By Lemma 4, the solution of ChrW can be obtained by calculating a circulation f on $\tilde{G}$ that minimizes $W(f)$. By Lemma 1, setting $u(e) = U(4|V|+1)(|E|+1)$ does not affect the solution. In addition, $|E_N| = O(|E|)$ and $|E_D| = O(|E|)$. Accordingly, the circulation f can be calculated in $O(|E|^2 \log |V| \log |E|)$ time by using Gabow's algorithm [20]. Therefore, the optimal solution can be calculated in $O(|E|^2 \log |V| \log |E|)$ time.

Proof of Theorem 3

ChrL is in NP because of Lemma 1.

Here, we show that the well-known PARTITION problem [34] can be reduced to ChrL. Let n be a positive integer and $S = \{i \in Z | 1 \leq i \leq n\}$. Also, let $s(i)$ be an integer function defined for $i \in S$ such that $s(i) > 0$, and $S_\Sigma = \sum_{i \in S} s(i)$. The problem of finding a subset $S' \subset S$ such that

$$\sum_{i \in S'} s(i) = \sum_{i \in S - S'} s(i) = S_\Sigma / 2$$

is called the *partition problem* (hereafter referred to as *PARTITION*) [34]. It is well known that PARTITION is NP-complete. We reduce PARTITION to ChrL by constructing a chromosome graph whose solution for ChrL contains two chromosomes that correspond to two subsets of a solution of PARTITION.

Let $G = (V, E)$ be a chromosome graph, where

$$V = \bigcup_{1 \leq i \leq n+1} \{v^-_{i,0}, v^+_{i,1}, v^-_{i,1}, v^+_{i,2}, v^-_{i,2}, v^+_{i,3}\}$$

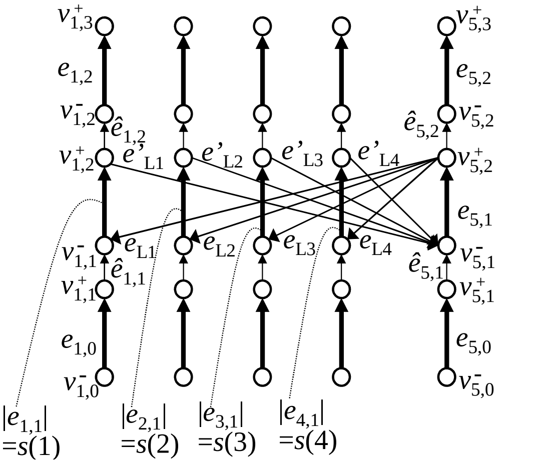

Figure 9 An example of a chromosome graph for solving the partition problem (PARTITION). In this example, $n = 4$.

is a set of vertices, and $E = E_S \cup E_L \cup E_R$ be a set of edges. Here, E_S consists of

$$\begin{aligned}
e_{i,0} &= \langle -v^-_{i,0}, +v^+_{i,1}, 1, 9S_\Sigma\rangle & (1 \le i \le n),\\
e_{i,1} &= \langle -v^-_{i,1}, +v^+_{i,2}, 2, s(i)\rangle & (1 \le i \le n),\\
e_{i,2} &= \langle -v^-_{i,2}, +v^+_{i,3}, 1, S_\Sigma - s(i)\rangle & (1 \le i \le n),\\
e_{n+1,0} &= \langle -v^-_{n+1,0}, +v^+_{n+1,1}, 2, 9S_\Sigma/2\rangle, &\\
e_{n+1,1} &= \langle -v^-_{n+1,1}, +v^+_{n+1,2}, n+2, 0\rangle, &\\
e_{n+1,2} &= \langle -v^-_{n+1,2}, +v^+_{n+1,3}, 2, 5S_\Sigma\rangle. &
\end{aligned}$$

In addition, E_R consists of

$$\begin{aligned}
\hat{e}_{i,1} &= \langle -v^+_{i,1}, +v^-_{i,1}, 0, 0\rangle \quad (1 \le i \le n{+}1),\\
\hat{e}_{i,2} &= \langle -v^+_{i,2}, +v^-_{i,2}, 0, 0\rangle \quad (1 \le i \le n{+}1),
\end{aligned}$$

and E_L consists of

$$\begin{aligned}
e_{Li} &= \langle +v^-_{i,1}, -v^+_{n+1,2}, 0, 0\rangle \quad (1 \le i \le n),\\
e'_{Li} &= \langle -v^+_{i,2}, +v^-_{n+1,1}, 0, 0\rangle \quad (1 \le i \le n).
\end{aligned}$$

We set $\lambda_i = 10S_\Sigma$ for any $i \ge 1$, $Q_N = Q_T = 100S_\Sigma$, $n_N = n+2$, and $n_T = 0$. See Figure 9 for an example. In addition, we set V_W to $V_5 \cup V_3$, and E_W to E by making all edges required so that G satisfies WCC.

We show that PARTITION for S has a solution $S' \subset S$ if, and only if, there exists a solution C of ChrL such that $W(C) = 0$. First, suppose that PARTITION has a solution S'. Let $r_{S'}$ be a cycle generated by merging cycles $e_{n+1,1}e_{Li}e_{i,1}e'_{Li}$ for $i \in S'$. We define $r_{S-S'}$ in the same way. Consider a multi-set $C = \{c_1, \ldots, c_{n+2}\}$, where $c_i \in C$ is a chromosome on G such that

$$\begin{aligned}
c_i &= e_{i,0}\hat{e}_{i,1}e_{i,1}\hat{e}_{i,2}e_{i,2} \;\; (1 \le i \le n),\\
c_{n+1} &= e_{n+1,0}\hat{e}_{n+1,1}r_{S'}e_{n+1,1}\hat{e}_{n+1,2}e_{n+1,2},\\
c_{n+2} &= e_{n+1,0}\hat{e}_{n+1,1}r_{S-S'}e_{n+1,1}\hat{e}_{n+1,2}e_{n+1,2}.
\end{aligned}$$

Then, $W(C) = 0$ because $|C| = n+2$, $\mathrm{Tr}(C) = 0$, and $m(C,e) = n(e)$ for $e \in E_S$. In addition, C visits all required edges. Furthermore, $|c_i| = 10\Sigma \le \lambda_i$ for $1 \le i \le n+2$.

Conversely, suppose that ChrL for G has an optimal solution C that satisfies $W(C) = 0$. Because $W(C) = 0$, we obtain $|C| = n+2$, $\mathrm{Tr}(C) = 0$, and $m(C,e) = n(e)$ for $e \in E$. Because $\sum_{e \in E} |e| n(e) = 10(n+2)S_\Sigma$, $|c| = 10\Sigma$ for each $c \in C$. Let c_i be a chromosome that begins with $e_{i,0}$ for $1 \le i \le n$. The other two chromosomes are denoted by c_{n+1} and c_{n+2}. Then, c_1 begins with $e_{1,0}\hat{e}_{1,1}e_{1,1}$. Suppose that c_1 does not visit $\hat{e}_{1,2}e_{1,2}$. Then, there is a chromosome c_i that visits $\hat{e}_{1,2}e_{1,2}$, whose previous edge has to be $e_{1,1}$ in c_i. Therefore, for some paths p_1 and p_2,

$$\begin{aligned}
c_1 &= e_{1,0}\hat{e}_{1,1}e_{1,1}p_1 \\
c_i &= p_2 e_{1,1}\hat{e}_{1,2}e_{1,2}.
\end{aligned} \tag{7}$$

Because of (7), $|c_1| = |e_{1,0}| + |\hat{e}_{1,1}| + |e_{1,1}| + |p_1| = 10S_\Sigma = |e_{1,0}| + |\hat{e}_{1,1}| + |e_{1,1}| + |\hat{e}_{1,2}| + |e_{1,2}|$. Therefore, $|p_1| = |\hat{e}_{1,2}| + |e_{1,2}|$. We modify C so that

$$\begin{aligned}
c_1 &= e_{1,0}\hat{e}_{1,1}e_{1,1}\hat{e}_{1,2}e_{1,2},\\
c_i &= p_2 e_{1,1} p_1.
\end{aligned}$$

The modified C still satisfies the required conditions. After this modification is repeated for $2 \le i \le n$ until no more modifications can be applied, C satisfies $c_i = e_{i,0}\hat{e}_{i,1}e_{i,1}\hat{e}_{i,2}e_{i,2}$ for $1 \le i \le n$. Another chromosome exists that visits $e_{i,1}$ for each $1 \le i \le n$, which is one of c_{n+1} and c_{n+2}. Let $S' = \{i | m(c_{n+1}, e_{i,1}) > 0\}$. Then, $\sum_{i \in S'} s(i) = 10S_\Sigma - (9/2+5)S_\Sigma = 1/2S_\Sigma$. Therefore, S' is a solution of PARTITION.

Competing interests
The authors declare that they have no competing interests.

Author's contributions
TY formulated the problem, proved the NP-completeness, developed a polynomial-time algorithm, and composed the manuscript. SM critically revised the manuscript.

Declarations
TY would like to acknowledge financial support from the Human Genome Center, Institute of Medical Science, University of Tokyo. This article has been published as part of *BMC* Genomics Volume 16 Supplement 2, 2015: Selected articles from the Thirteenth Asia Pacific Bioinformatics Conference (APBC 2015): Genomics. The full contents of the supplement are available online at http://www.biomedcentral.com/bmcgenomics/supplements/16/S2

Author details
[1]The Human Genome Center, Institute of Medical Science, University of Tokyo, Shiroganedai, Minato-ku, Tokyo, JP. [2]Department of Computer Science, University of Tokyo, Hongo, Bunkyo-ku, Tokyo, JP.

References

1. DNA Sequencing Costs. http://www.genome.gov/sequencingcosts/
2. Weischenfeldt, J., Symmons, O., Spitz, F., Korbel, J.O.: Phenotypic impact of genomic structural variation: insights from and for human disease. Nat Rev Genet **14**(2), 125–138 (2013)
3. Bashir, A., Volik, S., Collins, C., Bafna, V., Raphael, B.J.: Evaluation of paired-end sequencing strategies for detection of genome rearrangements in cancer. PLoS Comput Biol **4**(4), 1000051 (2008)
4. Campbell, P.J., Stephens, P.J., Pleasance, E.D., O'Meara, S., Li, H., Santarius, T., Stebbings, L.A., Leroy, C., Edkins, S., Hardy, C., Teague, J.W., Menzies, A., Goodhead, I., Turner, D.J., Clee, C.M., Quail, M.A., Cox, A., Brown, C., Durbin, R., Hurles, M.E., Edwards, P.A.W., Bignell, G.R., Stratton, M.R., Futreal, P.A.: Identification of somatically acquired rearrangements in cancer using genome-wide massively parallel paired-end sequencing. Nat Genet **40**(6), 722–9 (2008)
5. Malhotra, A., Lindberg, M.R., Faust, G.G., Leibowitz, M.L., Clark, R.A., Layer, R., Quinlan, A.R., Hall, I.M.: Breakpoint profiling of 64 cancer genomes reveals numerous complex rearrangements spawned by homology-independent mechanisms. Genome Res **23**(5), 762–776 (2013)
6. Stephens, P.J., Greenman, C.D., Fu, B., Yang, F., Bignell, G.R., Mudie, L.J., Pleasance, E.D., Lau, K.W., Beare, D., Stebbings, L.A., *et al.*: Massive genomic rearrangement acquired in a single catastrophic event during cancer development. Cell **144**(1), 27–40 (2011)
7. Shen, M.M.: Chromoplexy: A new category of complex rearrangements in the cancer genome. Cancer Cell **23**(5), 567–569 (2013)
8. Baca, S.C., Prandi, D., Lawrence, M.S., Mosquera, J.M., Romanel, A., Drier, Y., Park, K., Kitabayashi, N., MacDonald, T.Y., Ghandi, M., Van Allen, E., Kryukov, G.V., Sboner, A., Theurillat, J.-P., Soong, T.D., Nickerson, E., Auclair, D., Tewari, A., Beltran, H., Onofrio, R.C., Boysen, G., Guiducci, C., Barbieri, C.E., Cibulskis, K., Sivachenko, A., Carter, S., Saksena, G., Voet, D., Ramos, A., Winckler, W., et al.: Punctuated evolution of prostate cancer genomes. Cell **153**(3), 666–677 (2013)
9. Kloosterman, W.P., Tavakoli-Yaraki, M., van Roosmalen, M.J., van Binsbergen, E., Renkens, I., Duran, K., Ballarati, L., Vergult, S., Giardino, D., Hansson, K., Ruivenkamp, C.A.L., Jager, M., van Haeringen, A., Ippel, E.F., Haaf, T., Passarge, E., Hochstenbach, R., Menten, B., Larizza, L., Guryev, V., Poot, M., Cuppen, E.: Constitutional chromothripsis rearrangements involve clustered double-stranded DNA breaks and nonhomologous repair mechanisms. Cell Rep **1**(6), 648–55 (2012)
10. Mills, R.E., Walter, K., Stewart, C., Handsaker, R.E., Chen, K., Alkan, C., Abyzov, A., Yoon, S.C., Ye, K., Cheetham, R.K., Chinwalla, A., Conrad, D.F., Fu, Y., Grubert, F., Hajirasouliha, I., Hormozdiari, F., Iakoucheva, L.M., Iqbal, Z., Kang, S., Kidd, J.M., Konkel, M.K., Korn, J., Khurana, E., Kural, D., Lam, H.Y.K., Leng, J., Li, R., Li, Y., Lin, C.-Y., Luo, R., et al., 1000 genomes project: Mapping copy number variation by population-scale genome sequencing. Nature **470**(7332), 59–65 (2011)
11. Medvedev, P., Stanciu, M., Brudno, M.: Computational methods for discovering structural variation with next-generation sequencing. Nat Methods **6**, 13–20 (2009)
12. Chen, K., Wallis, J.W., McLellan, M.D., Larson, D.E., Kalicki, J.M., Pohl, C.S., McGrath, S.D., Wendl, M.C., Zhang, Q., Locke, D.P., Shi, X., Fulton, R.S., Ley, T.J., Wilson, R.K., Ding, L., Mardis, E.R.: BreakDancer: an algorithm for high-resolution mapping of genomic structural variation. Nat Methods **6**(9), 677–681 (2009)
13. Hormozdiari, F., Alkan, C., Eichler, E.E., Sahinalp, S.C.: Combinatorial algorithms for structural variation detection in high-throughput sequenced genomes. Genome Res **19**(7), 1270–1278 (2009)
14. Quinlan, A.R., Clark, R.A., Sokolova, S., Leibowitz, M.L., Zhang, Y., Hurles, M.E., Mell, J.C., Hall, I.M.: Genome-wide mapping and assembly of structural variant breakpoints in the mouse genome. Genome Res **20**(5), 623–635 (2010)
15. Abyzov, A., Urban, A.E., Snyder, M., Gerstein, M.: CNVnator: An approach to discover, genotype, and characterize typical and atypical CNVs from family and population genome sequencing. Genome Res **21**(6), 974–984 (2011)
16. Ye, K., Schulz, M.H., Long, Q., Apweiler, R., Ning, Z.: Pindel: a pattern growth approach to detect break points of large deletions and medium sized insertions from paired-end short reads. Bioinformatics **25**(21), 2865–2871 (2009)
17. Genome Reference Consortium. http://www.ncbi.nlm.nih.gov/projects/genome/assembly/grc/human/
18. Oesper, L., Ritz, A., Aerni, S., Drebin, R., Raphael, B.: Reconstructing cancer genomes from paired-end sequencing data. BMC Bioinformatics **13**(Suppl 6), 10 (2012)
19. Medvedev, P., Brudno, M.: Maximum likelihood genome assembly. J Comput Biol **16**(8), 1101–1116 (2009)
20. Gabow, H.N.: An efficient reduction technique for degree-constrained subgraph and bidirected network flow problems. In Proceedings of the 15-th annual ACM symposium on Theory of computing (STOC), 448–456 (1983)
21. Nagarajan, N., Pop, M.: Parametric complexity of sequence assembly: Theory and applications to next generation sequencing. J Comput Biol **16**(7), 897–908 (2009)
22. Li, R., Zhu, H., Ruan, J., Qian, W., Fang, X., Shi, Z., Li, Y., Li, S., Shan, G., Kristiansen, K., Li, S., Yang, H., Wang, J., Wang, J.: De novo assembly of human genomes with massively parallel short read sequencing. Genome Res **20**(2), 265–272 (2010)
23. Zerbino, D.R., Birney, E.: Velvet: Algorithms for de novo short read assembly using de bruijn graphs. Genome Res **18**, 821–829 (2008)
24. Simpson, J.T., Wong, K., Jackman, S.D., Schein, J.E., Jones, S.J.M., Birol, İ.: ABySS: A parallel assembler for short read sequence data. Genome Res **19**(6), 1117–1123 (2009)
25. Gnerre, S., MacCallum, I., Przybylski, D., Ribeiro, F.J., Burton, J.N., Walker, B.J., Sharpe, T., Hall, G., Shea, T.P., Sykes, S., Berlin, A.M., Aird, D., Costello, M., Daza, R., Williams, L., Nicol, R., Gnirke, A., Nusbaum, C., Lander, E.S., Jaffe, D.B.: High-quality draft assemblies of mammalian genomes from massively parallel sequence data. Proc Natl Acad Sci USA **108**(4), 1513–1518 (2011)
26. Kim, J., Larkin, D.M., Cai, Q., Asan, Zhang, Y., Ge, R.-L., Auvil, L., Capitanu, B., Zhang, G., Lewin, H.A., Ma, J.: Reference-assisted chromosome assembly. Proc Natl Acad Sci USA **110**(5), 1785–1790 (2013)
27. Pop, M.: Genome assembly reborn: recent computational challenges. Brief Bioinform **10**(4), 354–366 (2009)
28. Gaul, E., Blanchette, M.: Ordering partially assembled genomes using gene arrangements. In: Bourque, G., El-Mabrouk, N. (eds.) Comparative Genomics. Lecture Notes in Computer Science, vol. 4205, pp. 113–128. Springer, Germany (2006)
29. Navin, N., Kendall, J., Troge, J., Andrews, P., Rodgers, L., McIndoo, J., Cook, K., Stepansky, A., Levy, D., Esposito, D., Muthuswamy, L., Krasnitz, A., McCombie, W.R., Hicks, J., Wigler, M.: Tumour evolution inferred by single-cell sequencing. Nature **472**(7341), 90–94 (2011)
30. Jahani, S., Setarehdan, S.K.: Centromere and length detection in artificially straightened highly curved human chromosomes. International Journal of Biological Engineering **2**(5), 56–61 (2012)
31. Kasai, F., O'Brien, P.C.M., Ferguson-Smith, M.A.: Afrotheria genome; overestimation of genome size and distinct chromosome GC content revealed by flow karyotyping. Genomics **102**(5-6), 468–471 (2013)
32. Myers, E.W.: The fragment assembly string graph. Bioinformatics **21**, 79–85 (2005)
33. Sorge, M., van Bevern, R., Niedermeier, R., Weller, M.: A new view on rural postman based on Eulerian extension and matching. Journal of Discrete Algorithms **16**, 12–33 (2012)
34. Garey, M.R., Johnson, D.S.: Computers and Intractability : A Guide to the Theory of NP-Completeness. W.H. Freeman & Company, New York (1979)
35. Ahuja, R.K., Magnanti, T.L., Orlin, J.B.: Network Flows: Theory, Algorithms, and Applications. Prentice Hall, New Jersey (1993)
